# Knockdown of KIF15 suppresses proliferation of prostate cancer cells and induces apoptosis through PI3K/Akt signaling pathway

**DOI:** 10.1038/s41420-023-01625-5

**Published:** 2023-09-01

**Authors:** Hai Bi, Xiaofei Hou, Qiyang Shen, Zenan Liu, Xuehua Zhu, Lulin Ma, Jian Lu

**Affiliations:** 1 Department of Urology, Shanghai General Hospital, Shanghai Jiaotong University School of Medicine, 200080 Shanghai, China; 2 Department of Urology, Peking University Third Hospital, 49 North Garden Road, Haidian District, 100191 Beijing, People’s Republic of China; 3 Peking University Health Science Center, No. 38 Xueyuan Road, Haidian District, 100191 Beijing, People’s Republic of China; 4 Peking University Ninth School of Clinical Medicine, 10 Tieyi Road, Yangfangdian, Haidian District, 100038 Beijing, People’s Republic of China

**Keywords:** Prostate cancer, Translational research

## Abstract

Prostate cancer is one of the most common malignancies in men, which has been considered a public health threat. KIF15 is a kind of driver protein, and its abnormal expression is closely related to the occurrence and development of malignant tumors. The purpose of the study was to explore the significance and role of KIF15 in prostate cancer and to show some potential value for prostate cancer. Immunohistochemistry analysis showed that KIF15 was highly expressed in prostate cancer tissues, which was also positively correlated with T Infiltrate. The loss-of-function and gain-of-function assays based on prostate cancer cells indicated that the change in KIF15 expression could significantly affect cell proliferation, tumorigenesis, migration, and cell apoptosis. The inhibition of prostate cancer development by KIF15 knockdown was also assured in vivo. The Human Apoptosis Antibody Array showed that CD40L, cytoC, DR6, and p21 were up-regulated upon KIF15 knockdown, while IGF-I and Survivin were down-regulated. Moreover, the involvement of the PI3K/Akt pathway in the KIF15-mediated regulation of prostate cancer was preliminarily proved. In summary, KIF15 was identified to play an important role in the development or biological progress of prostate cancer and is considered to possess the potential to be used as a therapeutic target.

## Introduction

Prostate cancer is one of the most common malignancies in men [1–3]. In recent years, the mortality of prostate cancer patients in China has been increasing year by year. The aging population is one of China’s current national conditions, and the incidence and mortality of prostate cancer patients in China will be in a period of high growth in the future [4]. Currently, although the prognosis of early prostate cancer patients is good, the prognosis of advanced patients is very poor, and the treatment methods are limited. Moreover, due to the concealment of prostate cancer and the low popularity of early screening for prostate cancer in China, there is still a large number of prostate cancer patients of whose tumors are in the advanced stage when diagnosed. Therefore, there is an urgent need to explore novel mechanisms and therapeutic targets for prostate cancer.

Kinesin is one of the motor proteins. It is a kind of protein that can use the energy released by ATP hydrolysis to generate thrust and carry out intracellular material transport or promote cellular movement. Kinesin not only participates in the transport of vesicles, organelles, chromosomes, protein complexes, and RNA to maintain the normal activity of cells, but also plays an important role in the activity of neurons, the development of the brain, and the improvement of memory [5, 6]. Kinesin Family member 15 (KIF15) is a member of the kinesin Superfamily, which can promote cell mitosis, participate in the mutual transport of intracellular substances, help the assembly of cell structure, conduct signals, and a series of life movements [7]. In recent years, a large number of studies have shown that abnormal expression or defects of kinesin can lead to the occurrence of diseases and even the formation of tumors. Some research groups have reported that the abnormal expression of KIF15 is related to the occurrence and development of a variety of malignancies [8–12]. However, the role of KIF15 in prostate cancer has not been reported.

Bearing all these in mind, our research group speculated that KIF15 may play an important role in the development and progression of prostate cancer. Therefore, we chose KIF15 as the research object to explore its possible influence on the progression of prostate cancer.

## Results

### The expression of KIF15 in prostate cancer tissues and normal prostate tissues

**Fig. 1:**
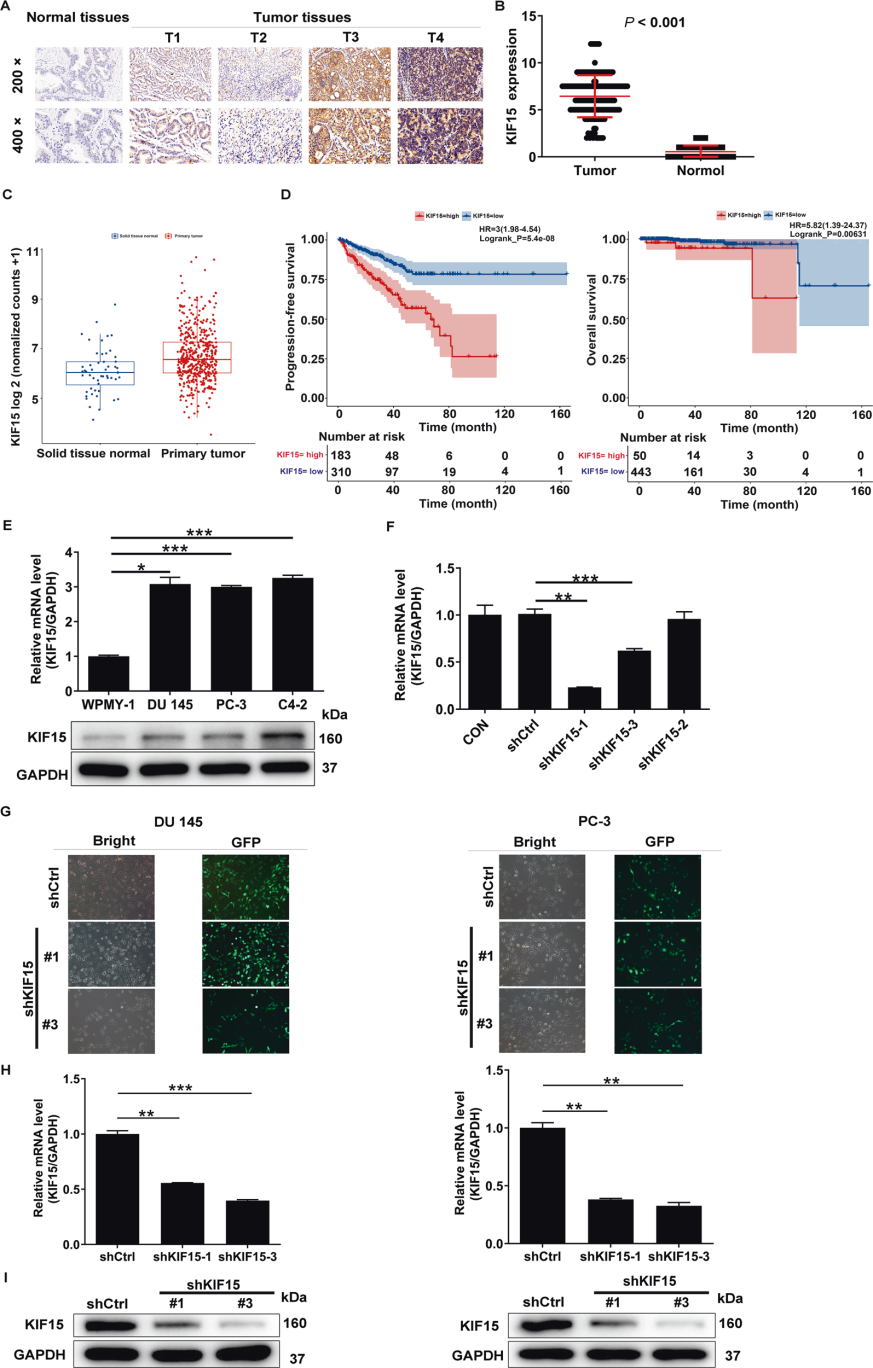
Preparation of human prostate cancer cells with KIF15 knockdown. **A** Detection of KIF15 expression in normal tissues and prostate cancer tissues with different pathological stages by IHC. **B** The IHC scores of normal tissues and prostate cancer tissues were collected and compared. **C** The differential expression of KIF15 in prostate cancer tissues and normal tissues was analyzed based on the data collected from the TCGA-PRAD dataset. **D** The correlation between KIF15 expression and prostate cancer patients’ prognosis (progression-free survival and overall survival) was analyzed based on the data collected from the TCGA-PRAD dataset. **E** The endogenous expression of KIF15 in immortalized cells of human normal prostate matrix WPMY-1 and human prostate cancer cell lines, including PC-3, DU 145, and C4-2, was detected by qPCR and western blot analysis, respectively. **F** The screening of more efficient shRNA was completed by qPCR. **G** The fluorescence of cells, which were infected with shCtrl or shKIF15 for 72 h, observed by microscope demonstrates a >80% efficiency of infection. H&I, The knockdown efficiencies of KIF15 in DU 145 and PC-3 cells were detected by qPCR (**H**) and western blot analysis (**I**). Data were shown as Mean with SD. **P < 0.01, ***P < 0.001.

First of all, the expression of KIF15 was detected in 155 cases of prostate cancer tissue and 79 cases of normal prostate tissue. We used the median of the half-quantified KIF15 expression score obtained from all tissue samples as a cut-off point for high and low KIF15 cases. Statistical analysis showed that the expression of KIF15 in prostate cancer tissues was significantly higher than that in normal prostate tissues (Fig. 1A, B and Table 1, *P* < 0.001). Although not statistically significant, immunohistochemical analysis showed that tumor tissues in more advanced pathological stages were accompanied by higher KIF15 expression (Fig. 1A). Moreover, according to Mann–Whitney *U*-analysis, there was a significant positive correlation between the high expression of KIF15 and the depth of tumor invasion (T Infiltrate), which was further confirmed in Spearman rank correlation analysis (Tables 2 and 3). Furthermore, data collected from the TCGA-PRAD dataset also indicated the upregulation of KIF15 in prostate cancer tissues compared with normal ones (Fig. 1C), as well as its negative correlation with patients’ overall survival and progression-free survival (Fig. 1D). Above all, the results showed that the expression of KIF15 increased with the deepening of tumor malignancy, indicating its potential role in tumor development.

**Table 1:** KIF15 expression pattern in prostate cancer tissues and normal prostate tissues revealed in immunohistochemistry analysis.

KIF15 expression	Tumor tissue		Normal tissue	
	Cases	Percentage	Cases	Percentage
Low	82	52.9%	79	100%
High	73	47.1%	0	-

*P* < 0.001.

**Table 2:** Relationship between KIF15 expression and tumor characteristics in patients with prostate cancer.

Features	No. of patients	KIF15 expression		*P*-value
		low	high	
All patients	155	82	73	
Age (years)				0.071
≤69	82	49	33	
>69	73	33	40	
T Infiltrate				0.034
T1	2	2	0	
T2	68	39	29	
T3	38	19	19	
T4	6	0	6	
Lymphatic metastasis (*N*)				0.106
N0	106	58	48	
N1	8	2	6	
Stage				0.066
I	14	8	6	
II	56	33	23	
III	32	17	15	
IV	12	2	10	
Gleason score				0.595
<8	60	32	28	
≥8	88	43	45

**Table 3:** Relationship between KIF15 expression and tumor characteristics in patients with prostate cancer.

		KIF15
T Infiltrate	Rs	0.199
	*P*-value	0.034
	N	114

### Preparation of human prostate cancer cells with KIF15 knockdown

Before constructing cell models, the endogenous expression of KIF15 in immortalized cells of human normal prostate matrix WPMY-1 and human prostate cancer cell lines, including PC-3, DU 145, and C4-2, was detected by qPCR and western blot analysis, respectively. The results showed that KIF15 expression was relatively higher in prostate cancer cells than in normal cells (Fig. 1E). According to the detection of KIF15 knockdown efficiency in DU 145 cells, the more efficient shRNA (shKIF15-1 and shKIF 15-3) was screened and used in the following experiments without further explanation (Fig. 1F). After the transfection of lentivirus in PC-3 and DU 145 cell lines, fluorescence imaging was performed to guarantee that transfection efficiency is higher than 80% (Fig. 1G). Afterward, the results of qPCR showed that the mRNA levels of KIF15 in DU 145 and PC-3 cells were effectively down-regulated by the transfection of shRNAs, respectively (Fig. 1H). The protein level of KIF15 was also distinctly suppressed after lentivirus transfection (Fig. 1I). The above experimental results indicated that DU 145 and PC-3 cells with KIF15 depletion were successfully prepared.

### Effects of down-regulation of KIF15 on prostate cancer cell phenotypes in vitro

**Fig. 2:**
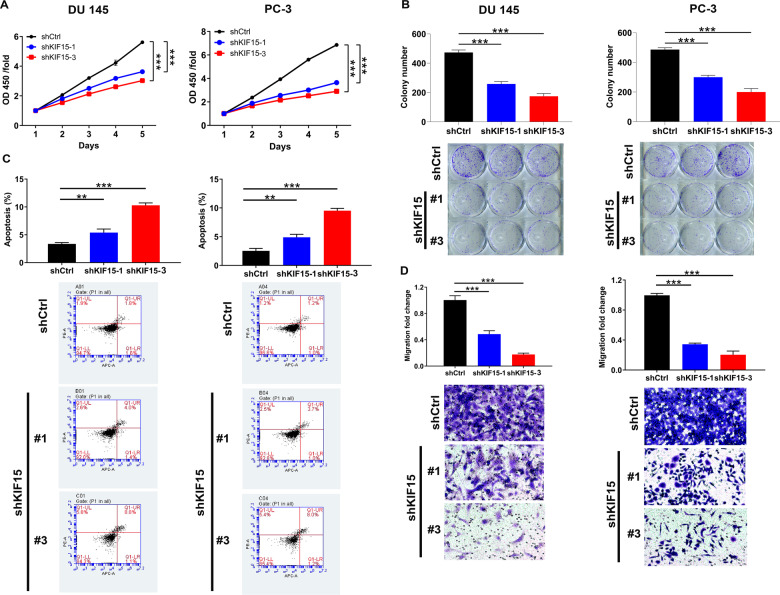
Knockdown of KIF15 inhibited prostate cancer development in vitro. **A** CCK8 assay was used to detect cell proliferation of DU 145 and PC-3 cells in shCtrl and shKIF15 groups with time from 24 h to 120 h. **B** After lentivirus infection of DU 145 cells and PC-3 cells, the number of colonies of shKIF15 groups and shCtrl group were counted and compared during 7 days of culturing. **C** 5 days after lentivirus infection, the apoptotic cell percentage of the shKIF15 group and shCtrl group was detected by flow cytometry. **D** Cell motility of DU 145 and PC-3 cells with or without KIF15 knockdown was detected by Transwell assay. Data were shown as mean with SD. **P < 0.01, ***P < 0.001.

CCK8 assay, flow cytometry, colony formation assay, and Transwell assay were used to evaluate the effects of down-regulation of KIF15 on prostate cancer phenotypes in vitro. The results of the CCK8 assay showed that, compared with the shCtrl group, knockdown of KIF15 slowed down the proliferation of DU 145 cells (*P* < 0.001, Fig. 2A) and PC-3 cells (*P* < 0.001, Fig. 2A), respectively. As expected, KIF15 depletion also decreased the number of the colonies formed by DU 145 or PC-3 cells (*P* < 0.001) (Fig. 2B). On the 5th day after lentivirus infection, the apoptosis rate of shCtrl group and shKIF15 groups was detected by flow cytometry to explore the effects of down-regulation of KIF15 on cell apoptosis of DU 145 and PC-3 cells, showing significantly enhanced cell apoptosis in shKIF15 group (*P* < 0.01) (Fig. 2C). Moreover, Transwell assays was performed to assess the effects of KIF15 on cell migration, which demonstrated that cell migration capability was strictly impeded after KIF15 knockdown (Fig. 2D). All these results provided evidence of the involvement of KIF15 in the development of prostate cancer.

### Effects of upregulation of KIF15 on prostate cancer cell phenotypes in vitro

**Fig. 3:**
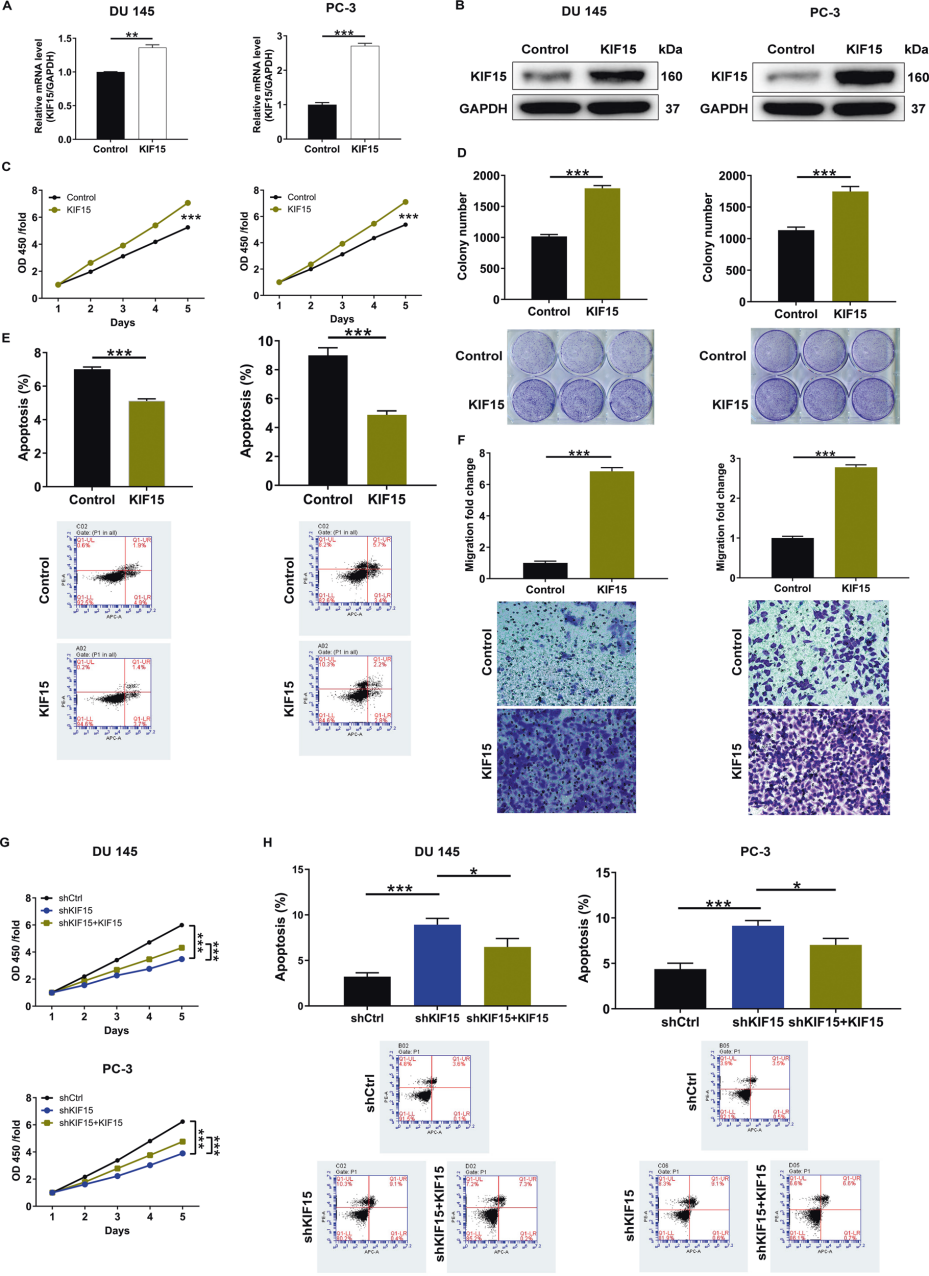
Overexpression of KIF15 promoted prostate cancer development in vitro. The overexpression efficiencies of KIF15 in DU 145 and PC-3 cells were detected by qPCR (**A**) and western blot analysis (**B**). **C** CCK8 assay was used to detect cell proliferation of DU 145 and PC-3 cells in Control and KIF15 groups with time from 24 h to 120 h. **D** After lentivirus infection of DU 145 cells and PC-3 cells, the number of colonies of the KIF15 group and Control group was counted and compared during 7 days of culturing. **E** 5 days after lentivirus infection, the apoptotic cell percentage of the KIF15 group and Control group was detected by flow cytometry. **F** Cell motility of DU 145 and PC-3 cells with or without KIF15 overexpression was detected by Transwell assay. Moreover, KIF15 knockdown cell models were transfected with KIF15 overexpression lentivirus for recovering KIF15 expression, which was subjected to the detection of cell proliferation (**G**) and cell apoptosis (**H**). Data were shown as Mean with SD. **P < 0.01, ***P < 0.001.

Except for the loss-of-function assays, the function of KIF15 in prostate cancer was further verified through gain-of-function assays. As shown in Fig. 3A, B, the overexpression of KIF15 in PC-3 cells was verified on mRNA and protein levels, respectively. Subsequently, the examination of prostate cancer cell phenotypes revealed completely opposite effects of KIF15 overexpression compared with KIF15 knockdown. The upregulation of KIF15 in prostate cancer cells accelerated cell proliferation (Fig. 3C), enhanced the formation of colonies (Fig. 3D), inhibited cell apoptosis (Fig. 3E), and promoted cell migration (Fig. 3F). More importantly, the recovery of KIF15 expression in KIF15 knockdown cells could alleviate the regulatory effects of KIF15 knockdown on cell proliferation and apoptosis (Fig. 3G, H). Collectively, the involvement of KIF15 in prostate cancer development was further proved.

### The exploration of the underlying mechanism of KIF15-induced regulation of prostate cancer

**Fig. 4:**
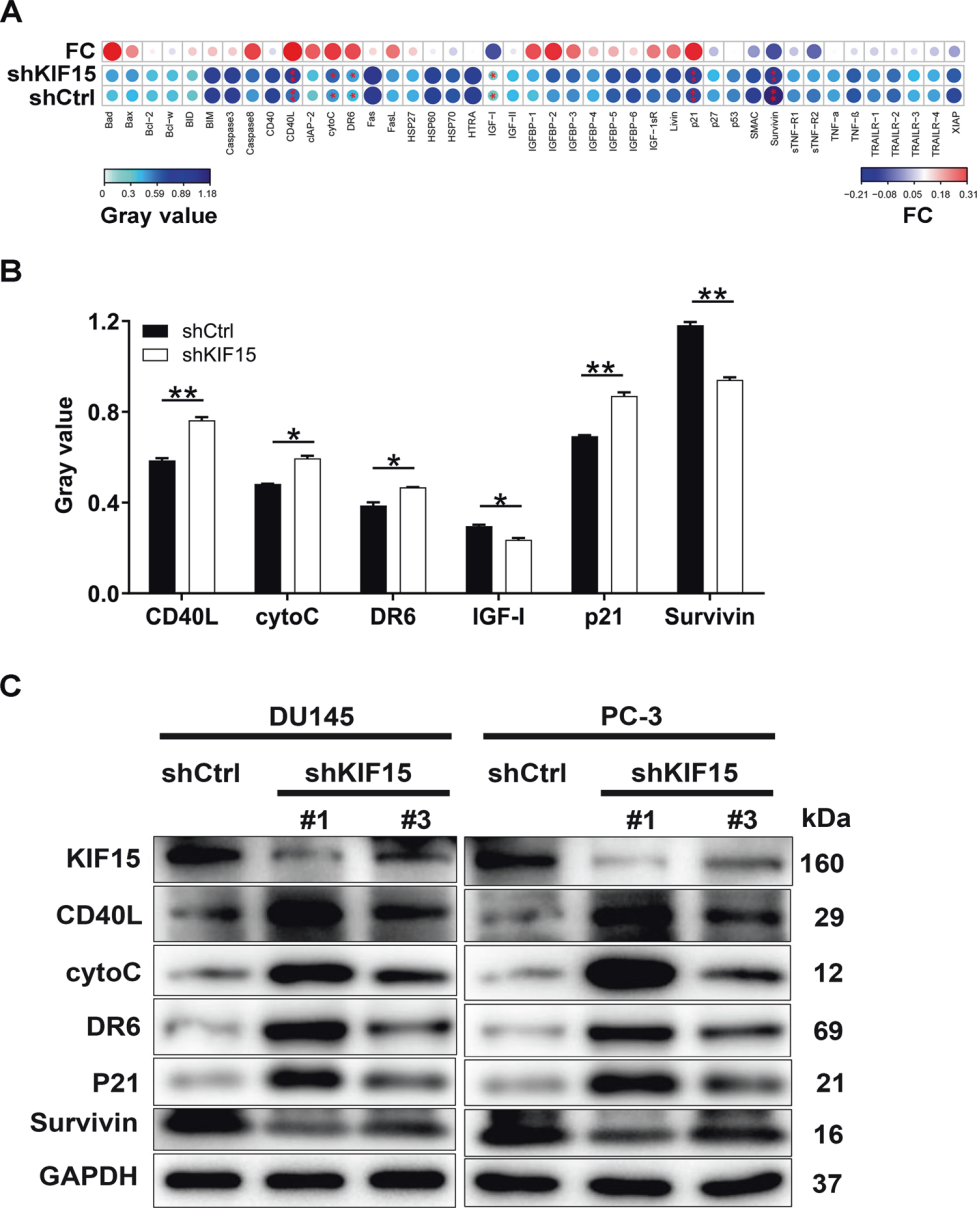
The underlying mechanism of KIF15 on prostate cancer was explored through antibody array and western blot analysis. **A** A Human Apoptosis Antibody Array was used to identify differentially expressed apoptosis-related proteins between shKIF15 and shCtrl groups of DU 145 cells. **B** The differentially expressed proteins identified by human apoptosis antibody array. **C** The protein levels of CD40L, cytoC, DR6, p21, and Survivin were detected by western blot analysis in DU 145 and PC-3 cells with or without KIF15 knockdown. Data were shown as mean with SD. *P < 0.05, **P < 0.01.

**Fig. 5:**
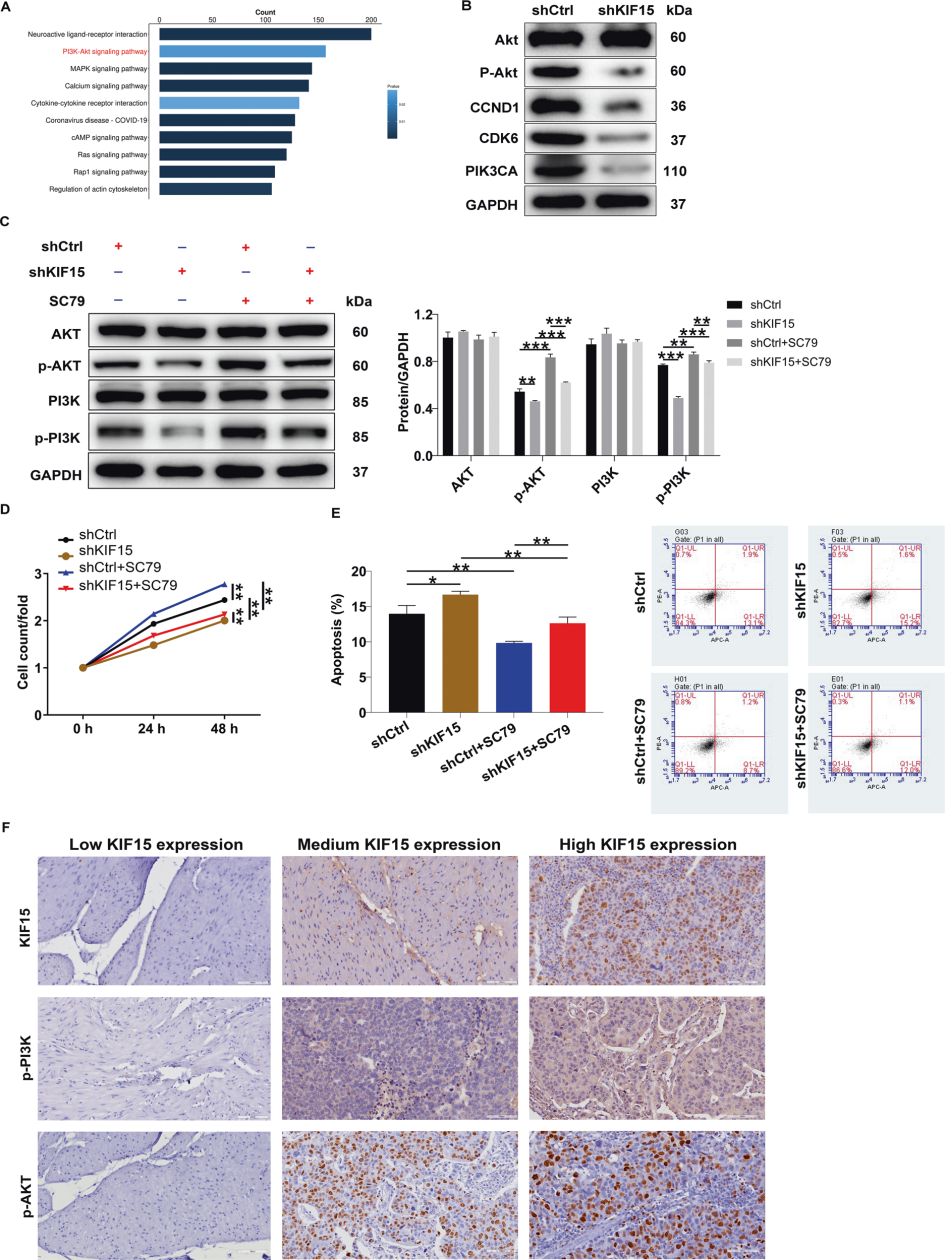
Knockdown of KIF15 may inhibit prostate cancer development through the PI3K/Akt signaling pathway. **A** A KEGG analysis was performed on genes with co-expression patterns with KIF15 obtained by coexpedia database. **B** The expression of several key proteins in the PI3K/Akt signaling pathway was detected in DU 145 cells with or without KIF15 knockdown. **C** The protein levels of Akt, p-Akt, PI3K, p-PI3K in shCtrl and shKIF15 DU 145 cells with or without treatment of Akt activator SC79 (10 μM) were detected by western blot analysis. D&E, The cell proliferation (**D**) and cell apoptosis (**E**) of shCtrl and shKIF15 DU 145 cells with or without treatment of Akt activator SC79 (10 μM) were detected by CCK8 assay and flow cytometry, respectively. **F** The expression of KIF15, p-PI3K, and p-Akt was detected in clinical specimens by IHC. Data were shown as mean with SD. *P < 0.05, ** *P* < 0.01.

A Human Apoptosis Antibody Array was performed to identify differentially expressed apoptosis-related proteins between shKIF15 and shCtrl groups of DU 145 cells. The results showed that CD40L, cytoC, DR6, and p21 were up-regulated in the shKIF15 group, while IGF-I and Survivin were down-regulated (*P* < 0.05) (Fig. 4A, B), which was further verified by western blot in both DU 145 and PC-3 cells (Fig. 4C). Moreover, we used coexpedia database (https://www.coexpedia.org/) to analyze the molecules possessing co-expression pattern with KIF15 and performed a KEGG signaling pathway enrichment analysis based on all co-expression factors of KIF15. As shown in Fig. 5A, the high enrichment of the PI3K/Akt pathway could be observed. Therefore, we further used western blot analysis to verify the effects of down-regulation of KIF15 on the expression of proteins related to the PI3k-Akt signaling pathway, which showed that CCND1, CDK6, and PIK3CA were down-regulated and phosphorylation of Akt was decreased (*P* < 0.05) (Fig. 5B). Moreover, upon the treatment of Akt pathway activator SC79, the suppressed expression of p-PI3K and p-Akt by KIF15 knockdown could be recovered to some extent, further verifying the regulatory effects of KIF15 on Akt pathway (Fig. 5C). Correspondingly, it was demonstrated that the inhibited cell proliferation (Fig. 5D) and strengthened cell apoptosis (Fig. 5E) induced by KIF15 knockdown could be partially rescued by SC79 treatment, indicative of the involvement of PI3K/Akt pathway in KIF15-mediated prostate cancer development. In addition, the relationship between KIF15 and PI3K/Akt pathway was also verified in clinical specimens. As shown in Fig. 5F, there was a high concordance between KIF15 expression and p-PI3K as well as p-Akt expression.

### Knockdown of KIF15 inhibited prostate cancer development in vivo

**Fig. 6:**
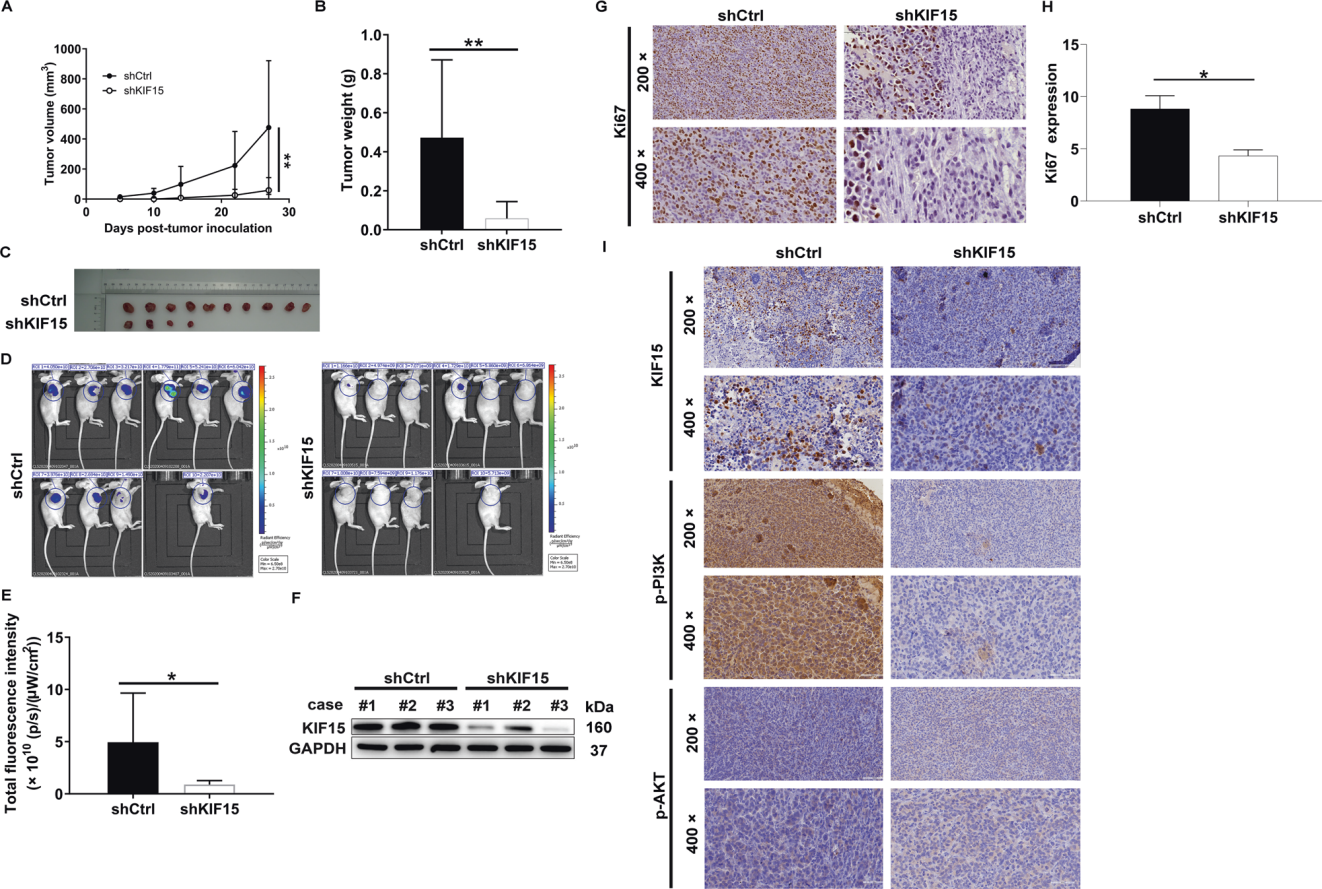
Knockdown of KIF15 inhibited tumor growth in vivo. **A** The tumor growth curve showed that the knockdown of KIF15 significantly inhibited tumor growth. **B** It could be observed from the removed xenografts that the volume of xenografts in the shKIF15 groups was distinctly smaller than that of the shCtrl group. **C** The weight of xenografts was obtained after the removal of xenografts. **D**, **E** Fluorescence imaging showed that the total fluorescence intensity in shKIF15 mice was significantly lower than that of the shCtrl group. **F** Compared with the shCtrl group, KIF15 protein expression in the shKIF15 group was significantly down-regulated. **G**–**I** After the collection of xenografts, a tumor section was prepared for the detection of Ki67, KIF15, p-PI3K, and p-Akt by IHC analysis. Data were shown as Mean with SD. *P < 0.05, **P < 0.01.

In vitro experiments showed that KIF15 had significant effects on the phenotypes of prostate cancer cells. To further verify its function, a mice xenograft model was constructed through subcutaneous injection of DU 145 cells with or without KIF15 silence. The results showed that the volume of the tumors formed in the shCtrl group was significantly larger than that of the shKIF15 group, and the difference between 2 groups increased over time (Fig. 6A). Also, the tumor weight of the shKIF15 group was significantly lighter compared with shCtrl group (*P* < 0.05) (Fig. 6B, C). Actually, similar results could also be observed through in vivo imaging, in which the intensity of fluorescence could indicate the growth of tumors in situ (Fig. 6D, E). Moreover, we also verified the expression of KIF15 in xenografts, which suggested a lower expression in shKIF15 groups (Fig. 6F). Finally, we sectioned the xenografts for immunohistochemistry to detect the effects of KIF15 on the expression of tumor proliferation index Ki67. The results showed that the Ki67 expression in the shKIF15 group was significantly lower than that in the shCtrl group (Fig. 6G, H). In addition, the down-regulation of KIF15 expression in shKIF15 xenografts was also accompanied by down-regulation of p-PI3K and p-Akt expression (Fig. 6I), which was in accordance with previous results obtained from prostate cell lines.

## Discussion

Based on epidemiological investigation, malignant tumor is still one of the main causes of death in human beings [13, 14]. However, the uncontrollability and unpredictability of malignant tumors have become a serious challenge for the prevention and treatment of malignant tumors [15]. The occurrence of malignant tumors is usually accompanied by abnormal expression of a variety of proteins. Various proteins with high or low expression are directly involved in various physiological and biochemical processes of cancer cells, such as enhancing drug resistance of cancer cells, enhancing proliferation and invasion ability of cancer cells, etc. Therefore, the exploration of novel, highly sensitive, and highly specific prostate cancer markers will contribute to the early diagnosis and treatment of prostate cancer [16].

The driver protein superfamily is encoded by more than 40 different human genes. In general, driver proteins are composed of four important domains, namely, n-terminal motor domain, stalk domain, connected motor domain, neck Linker domain, and Tail domain. Most of them move along microtubules in one direction, carrying molecules or organelles around the cells [17–19]. Abnormal expression or loss of kinesin can lead to the occurrence of diseases or even the formation of tumors. An in-depth understanding of its specific mechanism can effectively prevent the occurrence of diseases from the source or delay the progression of diseases.

A large number of studies have shown that abnormal expression of kinesin is closely related to the occurrence and development of some types of malignant tumors [20–22]. Research by Venere et al. [23] showed that interference of KIF11 in malignant gliomas by a small molecule blocker can inhibit the proliferation of glial tumor stem cells and normal glial tumor cells, while the upregulation of KIF11 in glial tumor stem cells can inhibit the expression of cdh1, promote cell cycle G1 / S transformation, accelerate the cancer stem cell proliferation rate. Ritter et al. [24] found that The S192 site of KIF2C (MCAK) can be phosphorylated by Aurora B (AURKB), which is an important kinase regulating the normal development of cell mitosis. Overexpression or differentiation can easily lead to abnormal mitosis, which is closely related to the growth and development of tumor cells. KIF15 is an important member of the driver protein, which is mainly involved in cell mitosis and nervous system development. Till now, the function of KIF15 in various types of human cancers has been reported, which was generally identified as a tumor promotor [9, 10, 12, 25–29].

In this study, we found a high expression of KIF15 in prostate cancer and demonstrated that the expression of KIF15 was positively correlated with T Infiltrate, which suggested the potential correlation between KIF15 and prostate cancer development. Moreover, we found that down-regulation of KIF15 expression significantly inhibited the proliferation of prostate cancer cells, as well as promoted prostate cancer cell apoptosis. Further, according to the results of colony formation and Transwell assay, we found that down-regulation of KIF15 expression could significantly inhibit the colony formation ability and mobility of prostate cancer cells. Correspondingly, overexpression of KIF15 in prostate cancer cells executed completely opposite regulatory effects. We then established a nude mouse model of subcutaneous tumor formation in vivo, and the results showed that interference with KIF15 expression could significantly inhibit the formation and growth of xenografts.

The expression of CD40L, cytoC, DR6, and P21 was significantly increased while the expression of IGF-I and Survivin was significantly decreased by down-regulating KIF15. Importantly, KEGG analysis showed that these altered proteins were all associated with the PI3K/Akt signaling pathway. What’s more important is that a lot of evidence has indicated that PI3K/Akt pathway is a key regulator in cancer cell apoptosis [30–32]. We therefore hypothesized that KIF15 regulates the PI3K/Akt signaling pathway to affect cell apoptosis. Further, we used WB to verify this proposal and found that the expressions of p-Akt, CCND1, CDK6, and PIK3CA were significantly decreased after the down-regulation of KIF15, which was consistent with the abovementioned results. Also, it was demonstrated that the change of PI3K/Akt pathway activity and cell phenotypes caused by KIF15 knockdown could be partially recovered by the treatment of the Akt pathway activator.

There are still some drawbacks in this study. The number of specimens included in this study is small. Moreover, further studies are needed to determine the efficacy of KIF15 as a prognostic or predictive factor. In the future, through further research on the molecular mechanism, we are expected to provide new methods for targeted therapy of prostate cancer, so that more prostate cancer patients can benefit.

## Conclusion

In this study, we found that down-regulation of KIF15 inhibited the proliferation, tumorigenesis, and migration of prostate cancer cells, as well as the subcutaneous tumor formation of prostate cancer cells through the PI3K/Akt pathway. KIF15 is highly expressed in prostate cancer tissues and is associated with poor prognosis. KIF15 may play an important role in the development of prostate cancer.

## Materials and methods

### Cell culture

Cells were purchased from ATCC and cultured in DMEM containing 10% fetal bovine serum and 1% penicillin in an incubator at 37 °C and 5% CO_2_. The cells were then digested with 0.25% trypsin and subcultured.

### Tissue microarray immunohistochemical staining

The 155-point prostate cancer tissue microarray (PR1921b) was purchased from Xi’an Alena Biotechnology Co., LTD., and the 79-point normal prostate tissue microarray (BNS19011) was purchased from Shaanxi Chaoying Biotechnology Co., LTD.

The tissue microarray was dewaxed with xylene and alcohol. Then, a citric acid antigen repair solution (Maixin) was used for antigen repair. The preparation method of citrate buffer solution was as follows: the powder was directly dissolved with ddH_2_O and diluted at a ratio of 1 packet /2000 ml ddH_2_O. After cooling to room temperature, endogenous peroxidase was blocked with 3% H_2_O_2_ for 5 min, washed with 1× PBST buffer solution, and sealed with 5% serum for 15 min. KIF15 antibody (FineTest, FNab04551), p-PI3K antibody (CST, 4228), and p-Akt antibody (Proteintech, 66444-1-Ig) were added and incubated at 4 °C overnight. After washing with 1× PBST buffer solution for 5 min/3 times, a secondary antibody was added for incubating overnight. DAB color development and hematosin redyeing were performed after washing with 1×PBST buffer solution for 5 min/3 times. Finally, the samples were dehydrated with alcohol, subjected to transparent xylene incubation, and sealed with neutral gum. A microscope was used for observation.

### Construction of plasmid vector and package of lentivirus

Multiple 19–21 nt RNA interference target sequences were designed with KIF15 as the gene template (reference sequence shCtrl was used as negative control), and GCTGAAGTGAAGAGGCTCAAA (human-KIF15-1), AGGCAGCTAGAATTGGAATCA (human-KIF15-2) and AAGCTCAGAAAGAGCCATGTT (human-KIF15-3) sequences were selected as the interference targets to construct the RNA interference lentivirus vector (shKIF15). Single-stranded DNA oligo-containing interference sequence was synthesized and double-stranded DNA was produced by annealing after the RNA interference target design was completed. The BR-V108 vector was linearized by AgeI and EcoRI double enzyme digestion, the double-stranded DNA oligo was attached to the linearized vector, and the ligation product was transformed into *Escherichia coli* receptive cells. The positive recombinants were identified by PCR and sent for sequencing verification. The sequencing results were compared to the correct clones for plasmid extraction for the next step of lentivirus packaging. Transfection of 293T cells with pHelper1.0, pHelper2.0 vector, and recombinant plasmid was performed 48–72 h after transfection (cell super plasmid). After centrifugation filtration, removal of impurities, lentivirus concentration was detected and purification was performed.

### Real-time qPCR detection

The total RNA was extracted according to Sigma’s Trizol operating instructions. RNA reverse transcription was performed and cDNA was obtained according to Vazyme Hiscript QRT Supermix for qPCR (+gDNA WIPER) operating instructions. The upstream primer sequence of KIF15 is CTCTCACAGTTGAATGTCCTTG, and the downstream primer sequence is CTCCTTGTCAGCAGAATGAAG. The upstream primer sequence of the internal reference gene GAPDH is TGACTTCAACAGCGACACCCA, and the downstream primer sequence is CACCCTGTTGCTGTAGCCAAA. After the PCR reaction solution was prepared, it was centrifuged instantaneously, and the centrifuged reaction solution was put into the PCR instrument for PCR amplification, and the experimental results were analyzed.

### Western blot assay

The cell culture medium was discarded. The cells were washed with PBS twice and an appropriate amount of pre-cooled 1× Lysis Buffer. The cells were fully lysed on ice for 10–15 min. Glue with different concentrations was prepared according to the molecular weight of the target protein. After adding the protein sample, electrophoresis was carried out, and the protein was transferred to the PVDF membrane. The PVDF membrane was sealed with TBST solution containing 5% skimmed milk and incubated overnight with 1:1000 diluted rabbit anti-KIF15 (Fine test, FNab04551), anti-CD40L (Abcam, ab65854), anti-cytoC (Abcam, ab13575), anti-DR6 (Proteintech, 66754-1-Ig), anti-p21 (Abcam, ab109520), anti-Survivin (Proteintech, 10508-1-AP) or 1:3000 diluted rabbit anti-GAPDH (Bioworld, AP0063). The membrane was soaked with TBST for 3 times /10 min. The PVDF membrane was incubated with 1: 3000 diluted HRP goat anti-rabbit IgG (Beyotime, A0208) at room temperature for 2 h. After soaking with TBST for 3 times/10 min, Amersham’s ECL+plusTM Western blotting system kit was used for color development.

### CCK8 assay

The CCK8 assay was used to assess cell viability and proliferation. Cells were seeded at a density of 5,000 cells per well in 96-well plates and incubated at 37 °C with 5% CO_2_ for 24 h to allow attachment. Various concentrations of test compounds or treatments were added and cells were incubated for specified time intervals. After treatment, 10 μl of CCK8 reagent was added to each well and incubated for an additional 2 h. Absorbance was measured at 450 nm using a microplate reader. Cell viability was determined by comparing the absorbance values of treated cells to control cells.

### Flow cytometry detection of cell apoptosis

Apoptosis was induced 5 days after lentivirus infection. The culture medium supernatant was collected in a 5 mL centrifuge tube and washed with PBS. After the cells were digested with trypsin, the digestion was stopped with culture medium supernatant, and the cells were collected in the same centrifuge tube. The cells were centrifuged at 1500 rpm for three times, during which the cell precipitation was washed with PBS and 1× binding buffer respectively. Cell precipitation was resuspended with a 1× cell staining buffer, then Annexin V-FITC (5 μL) and propidium iodide (PI, 20 μg/ml, 5 μl) were added to 100 μL cell suspension (1 × 10^5^ to 1 × 10^6^ cells) for staining. Then the supernatant was removed by centrifugation and the cells were resuspended. The detection is carried out by flow cytometry.

### Colony formation assay

After 5 days of lentivirus infection, a 6-well plate was planted with 500 cells per well. Three wells were set for each experimental group. The cells were cultured continuously for 7 days, and the medium was changed every 3 days during the process and the cell status was observed. 1 mL of 4% paraformaldehyde was added to each well. The cells were fixed for 30–60 min and were washed once with PBS. GIEMSA staining solution of 500 μL was added to each well for 10–20 min. The cells were washed several times with ddH_2_O and photographed and counted with a digital camera.

### Transwell assay

Cellular migration was also detected by using the Transwell kit (3422 Corning). Lentivirus-infected cells were distributed in 24-well plates at a density of 100,000 cells per well. The cells were isolated from the serum-containing medium using the Transwell chamber. Serum-free medium was added in the upper chamber and 30% FBS medium was added in the lower chamber. After incubation for 24 h, the nonmetastatic cells were gently removed with a cotton swab. The dye solution was added to an empty well of a 24-well plate for 20 min. The invasion of cells was pictured and counted with a microscope.

### Animal experiments

Twenty nude mice were selected and were divided into two groups. The first group was injected with DU145 cells infected with negative control lentivirus (shCtrl group), and the second group was injected with DU145 cells infected with KIF15 knockdown lentivirus (shKIF15 group). Tumor-forming cells were prepared according to experimental groups. The right front leg of each nude mouse was disinfected with alcohol for 3 times, and 200 μL cell suspension (4000000 cells/mouse) was injected subcutaneously. After injection, the needle was pulled out slowly for a while to prevent the cell suspension from leaking. All nude mice were cultured in the same conditions, that is, an SPF laminar flow chamber with a constant temperature (22 to 25 °C) and constant humidity (40 to 50%), alternating 12/12 h light and dark, standard sterile grain feed, free food, regular replacement of wood, one time/week observation to make corresponding records (quality, tumor size). After 7 weeks, the nude mice were sacrificed by cervical dislocation. The nude mice were dissected, and the tumors were removed and photographed. In vivo imaging was performed on the animals before they were killed: the nude mice were anesthetized by intraperitoneal injection of 0.7% pentobarbital sodium at 10 μl/g. The animals were placed in a living body imager for imaging, fluorescence observation, and data preservation.

### Statistical analysis

All experiments were performed in independent biological triplicate and data were shown as mean ± SD (*n* ≥ 3). Statistical analysis was performed by GraphPad Prism software 6.0 and SPSS (20.0) using unpaired Student’s *t*-test and one-way ANOVA followed by Bonferroni’s post hoc test. The Mann–Whitney *U*-analysis and Pearson correlation analysis were applied to explore the correlation between KIF15 expression and tumor features of patients. *P* < 0.05 indicated a significant difference.

### Supplementary information

Supplemental Materials

## Data Availability

The data that support the findings of this study are available from the corresponding author (Jian Lu) upon reasonable request.
