# Supplementary material for: Knockdown of KIF15 suppresses proliferation of prostate cancer cells and induces apoptosis through PI3K/Akt signaling pathway
Source: Cell Death Discov. 2023 Sep 1;9:326. doi: 10.1038/s41420-023-01625-5 (PMC10474048; doi:10.1038/s41420-023-01625-5)
Supplement: Supplementary file 1 — Supplemental Materials [file 41420_2023_1625_MOESM1_ESM.pdf]

Supplemental Material: The full length uncropped original western blots for Fig. 1E

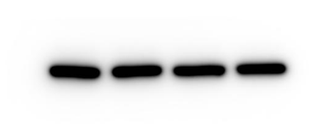

GAPDH

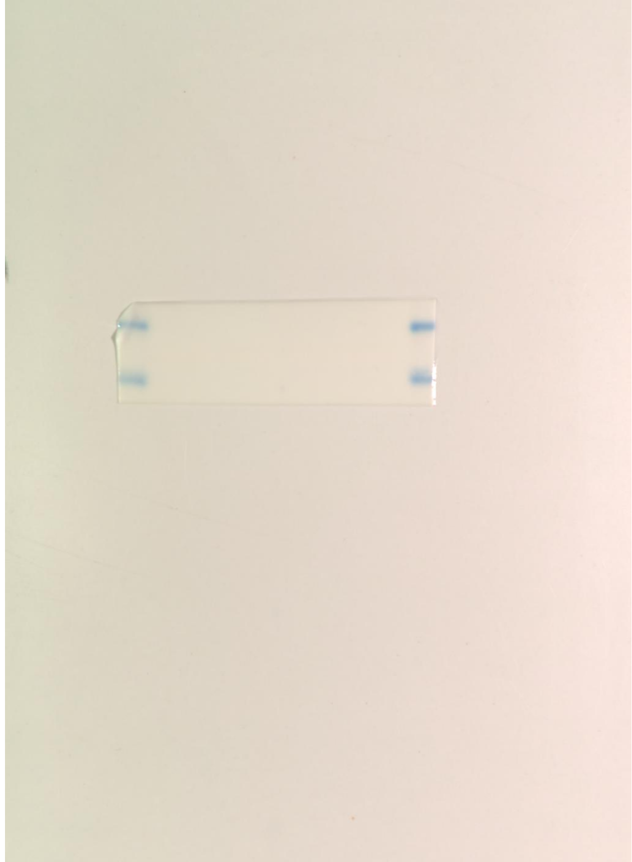

GAPDH M

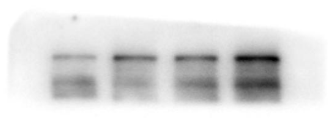

KIF15

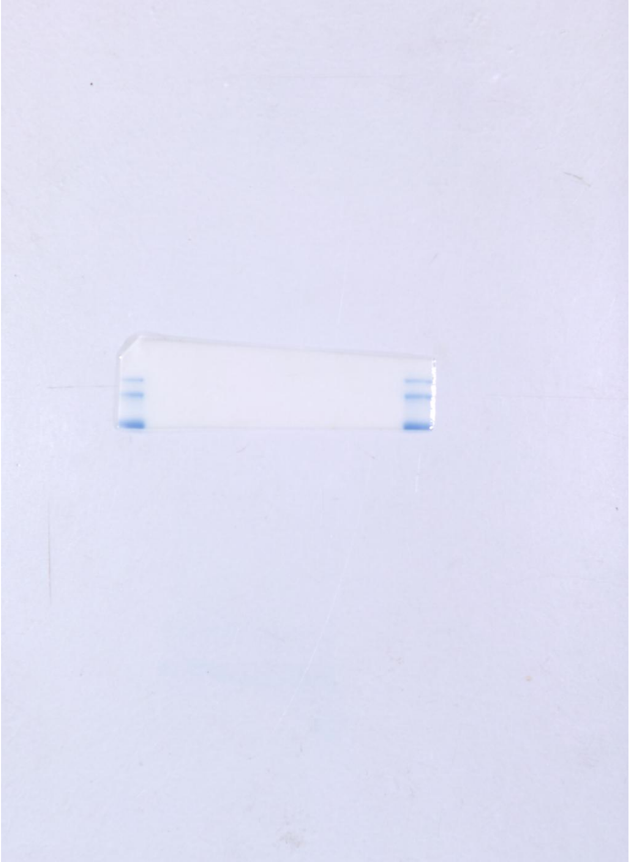

KIF15 M

Supplemental Material: The full length uncropped original western blots for Fig. 1I/DU 145

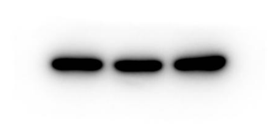

GAPDH

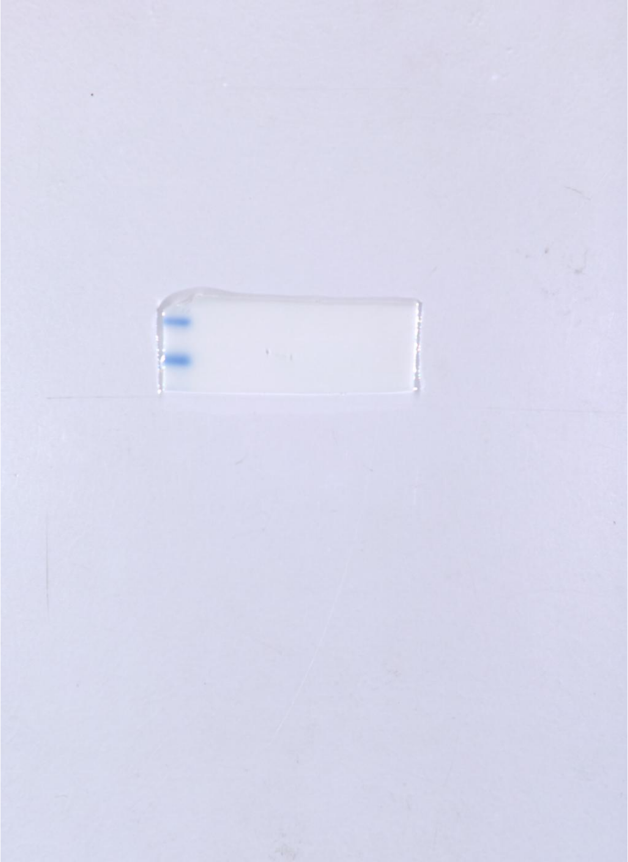

GAPDH M

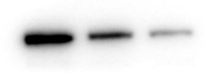

KIF15

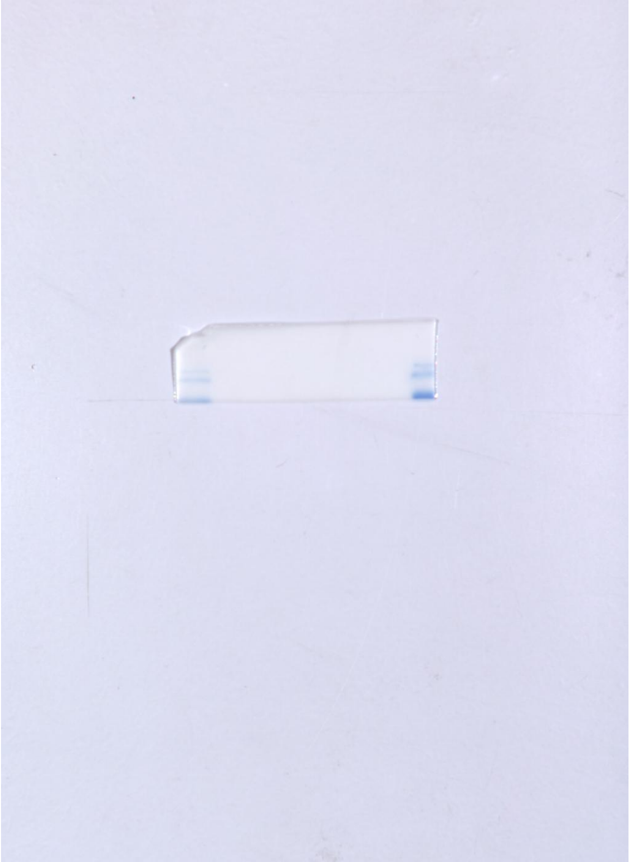

KIF15 M

Supplemental Material: The full length uncropped original western blots for Fig. 1I/PC-3

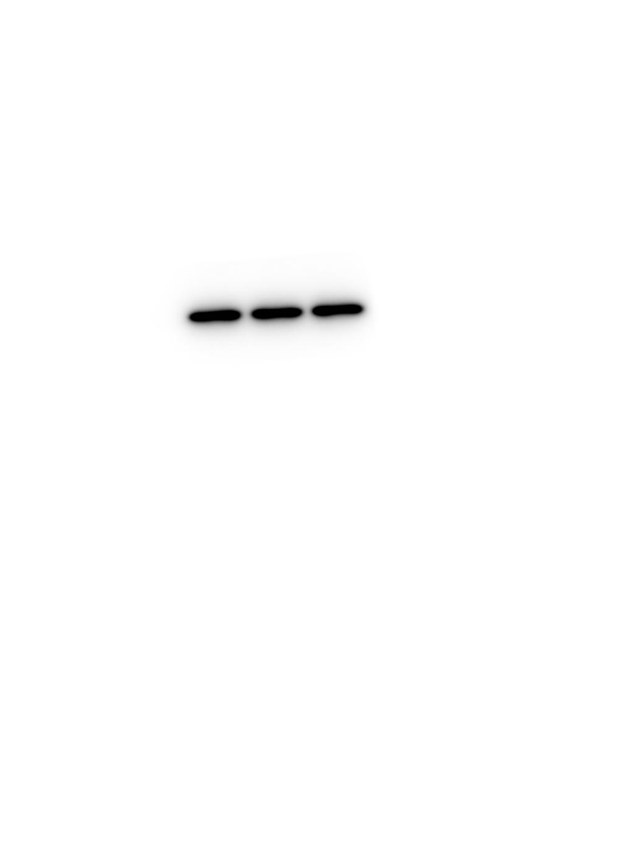

GAPDH

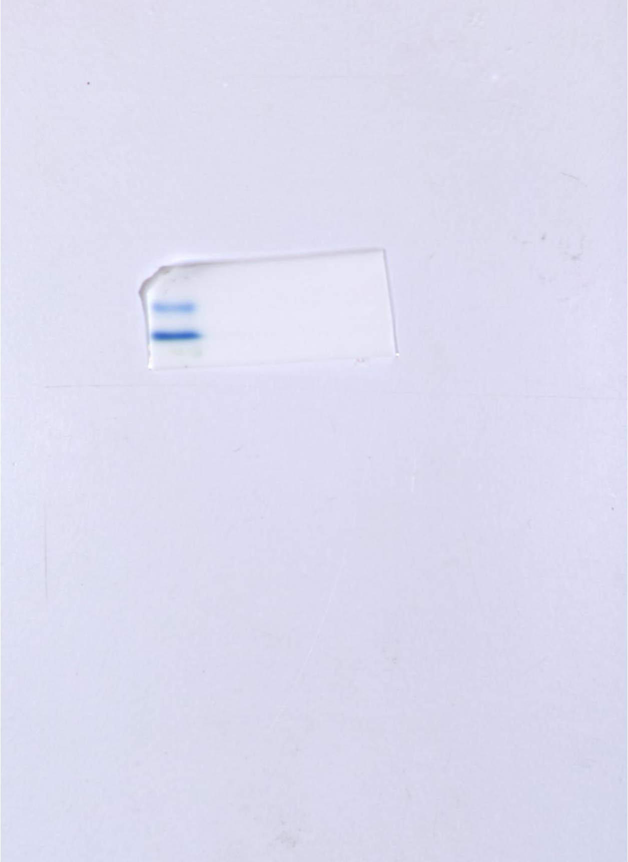

GAPDH M

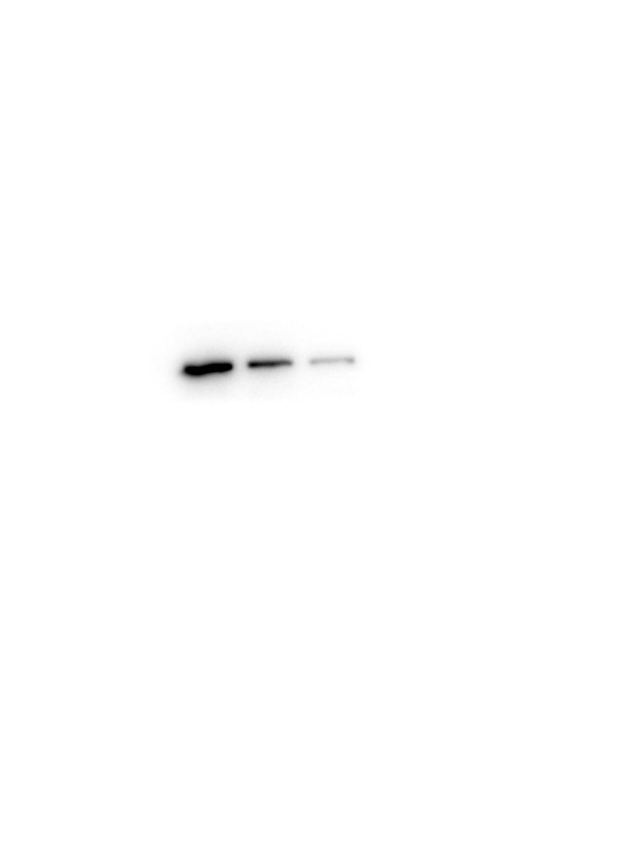

KIF15

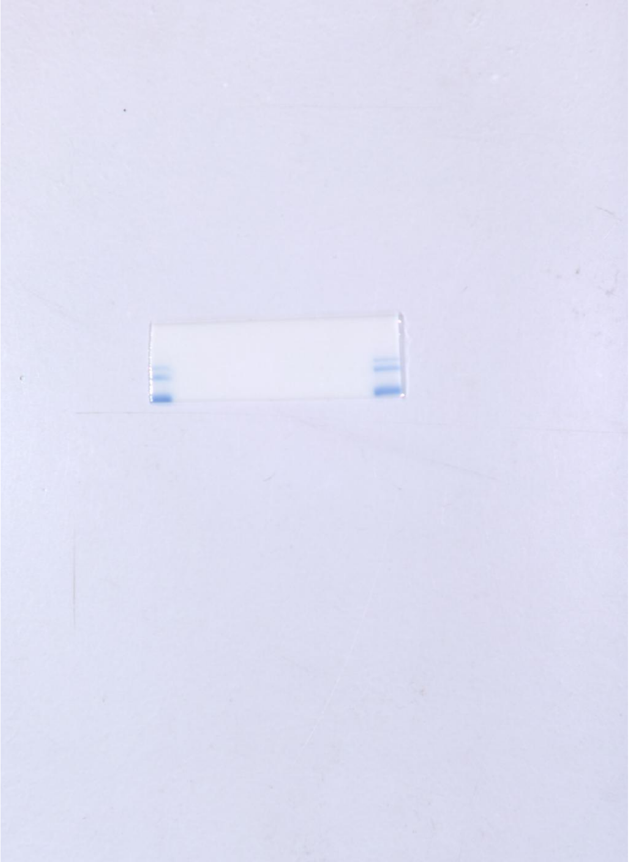

KIF15 M

Supplemental Material: The full length uncropped original western blots for Fig. 3B/DU 145

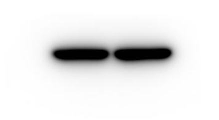

GAPDH

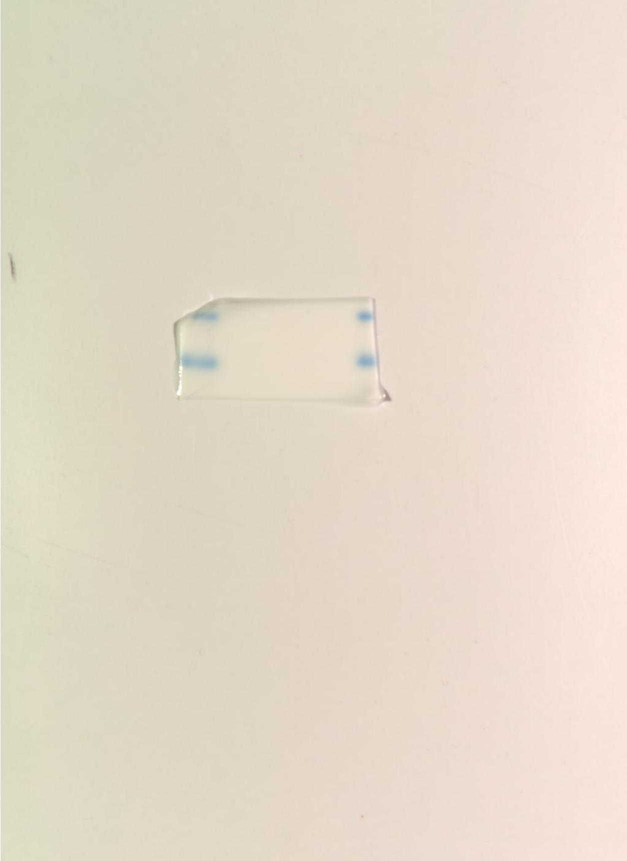

GAPDH M

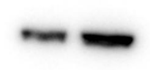

KIF15

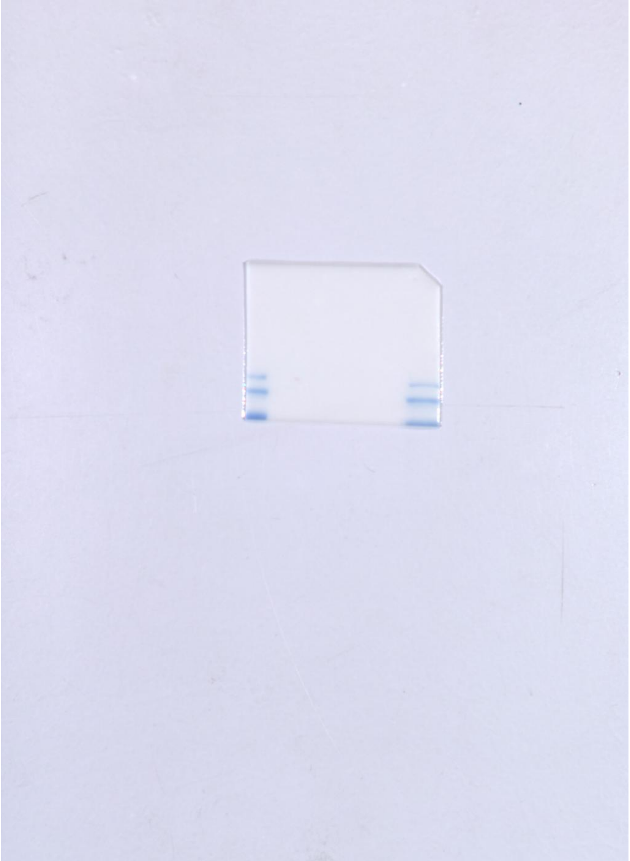

KIF15 M

Supplemental Material: The full length uncropped original western blots for Fig. 3B/PC-3

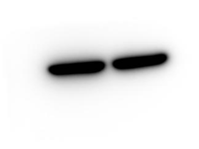

GAPDH

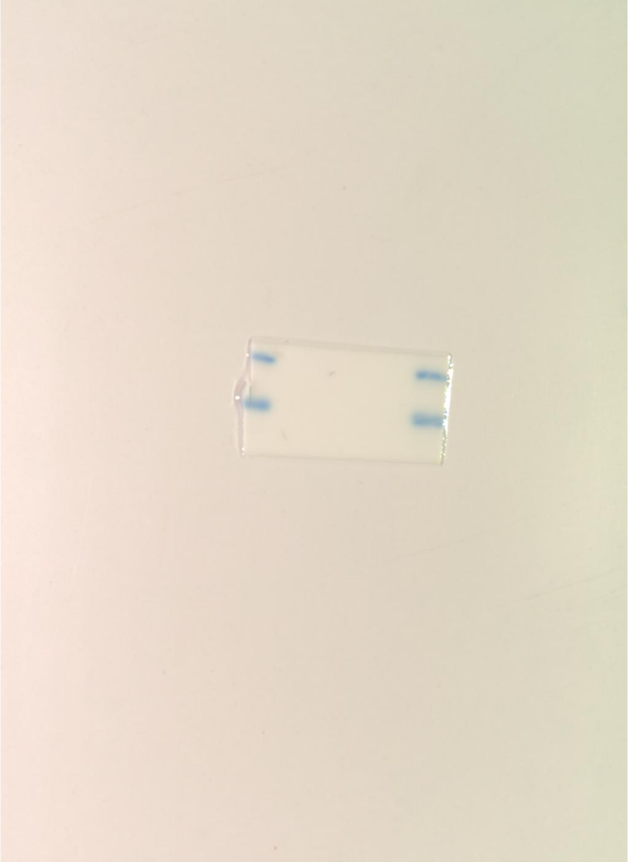

GAPDH M

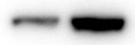

KIF15

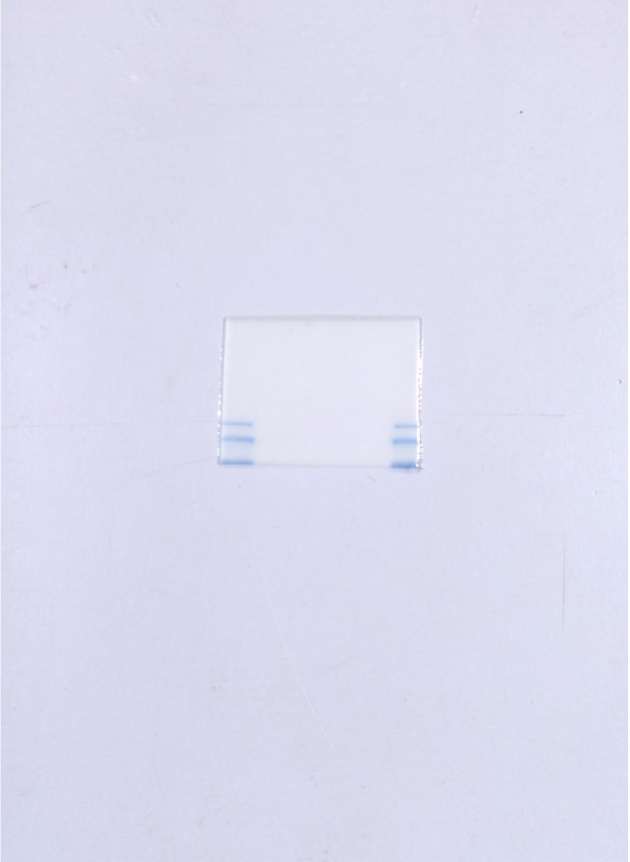

KIF15 M

Supplemental Material: The full length uncropped original western blots for Fig. 4C/DU 145

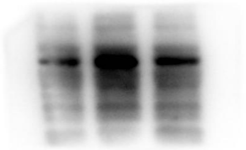

CD40L

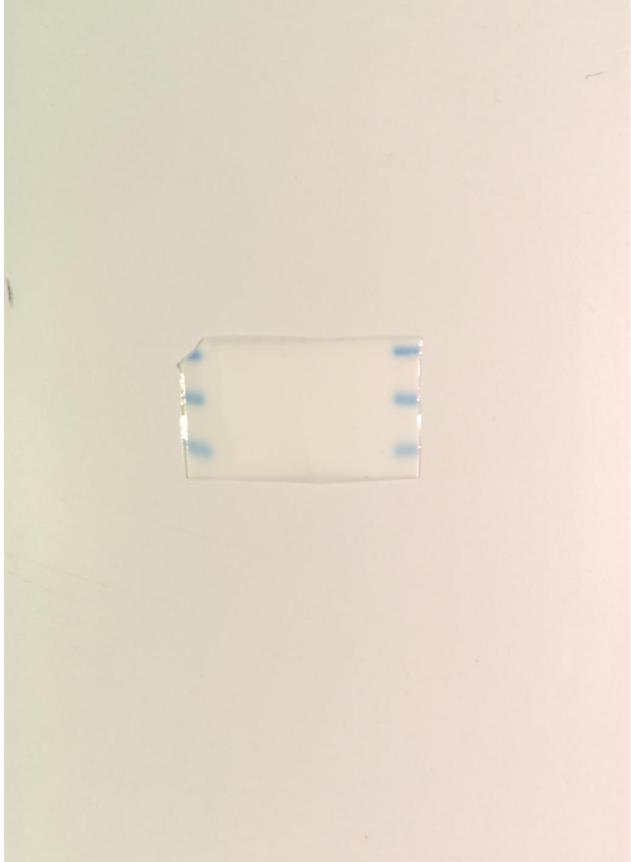

CD40L M

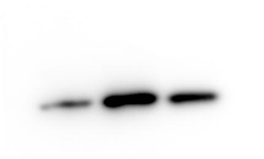

CYTC

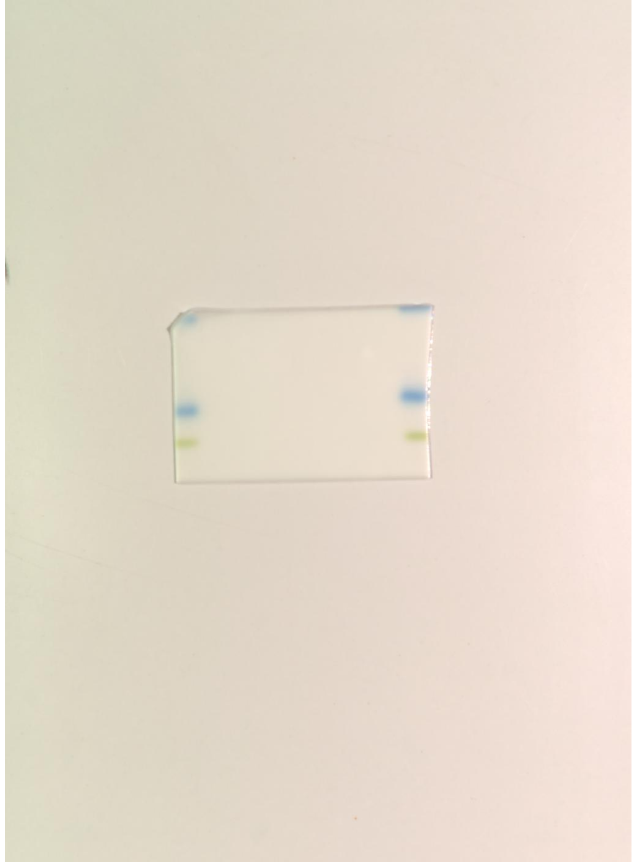

CYTC M

Supplemental Material: The full length uncropped original western blots for Fig. 4C/DU 145

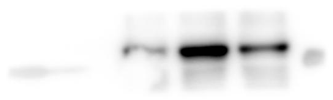

DR6

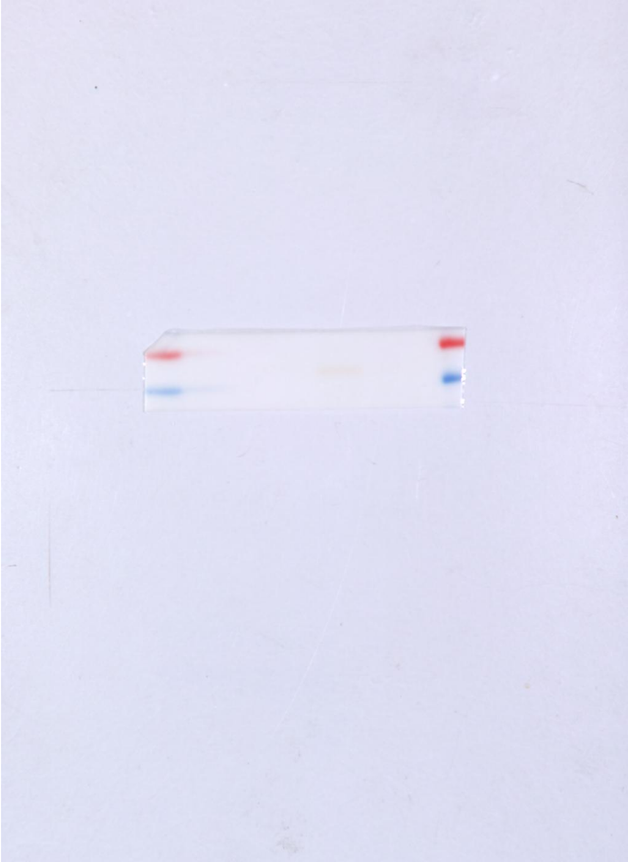

DR6 M

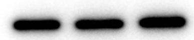

GAPDH

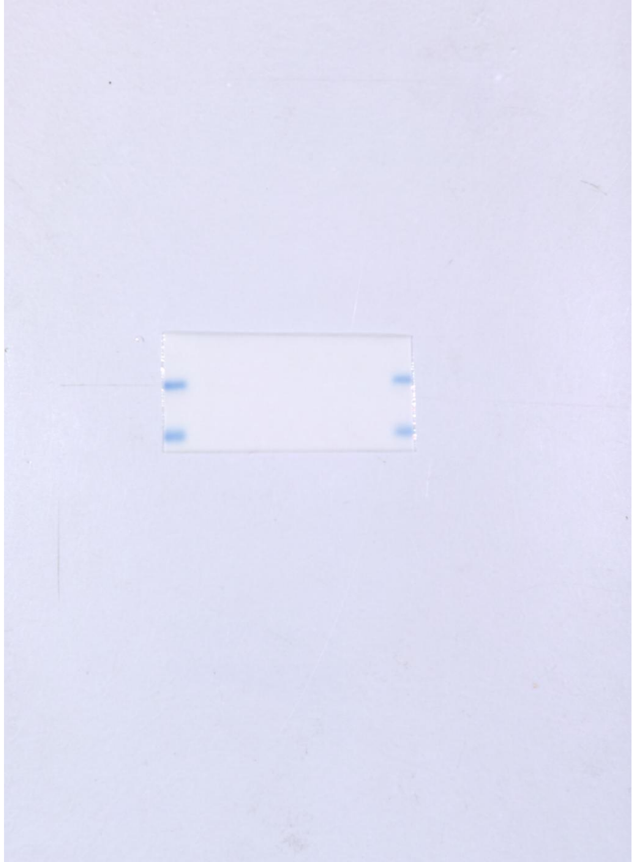

GAPDH M

Supplemental Material: The full length uncropped original western blots for Fig. 4C/DU 145

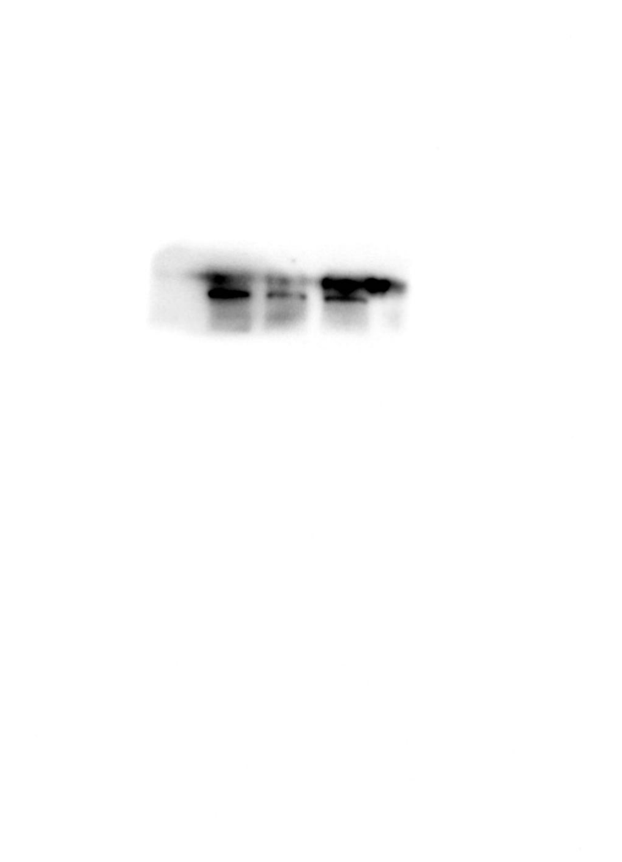

KIF15

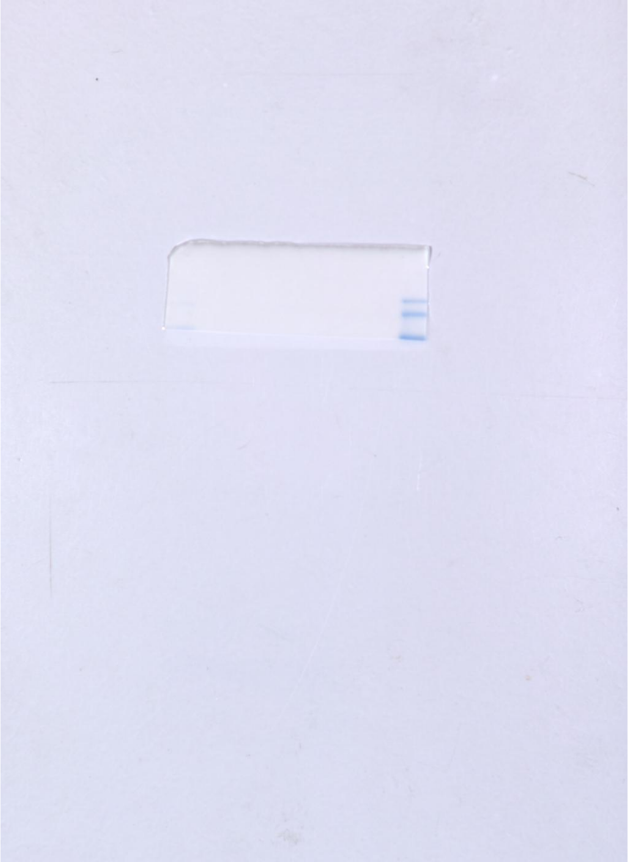

KIF15 M

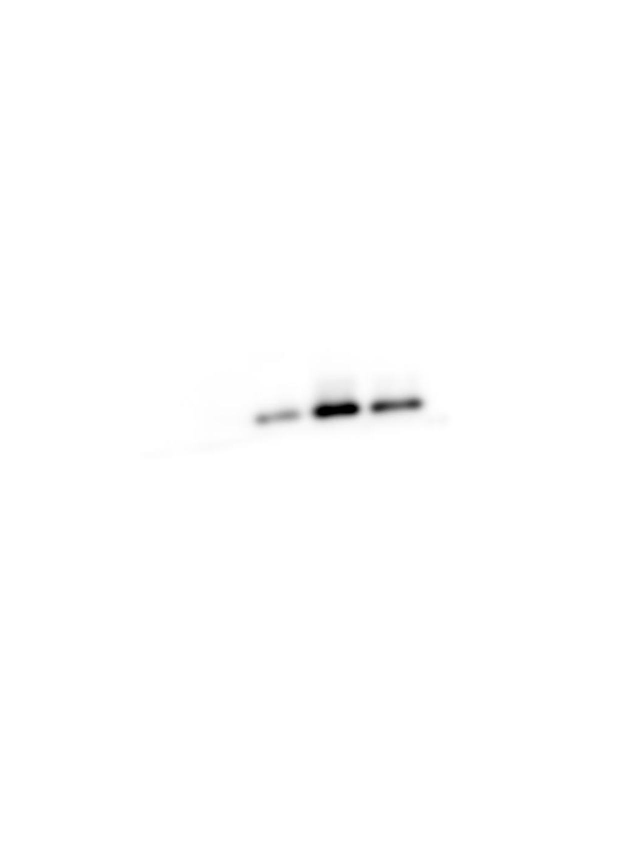

P21

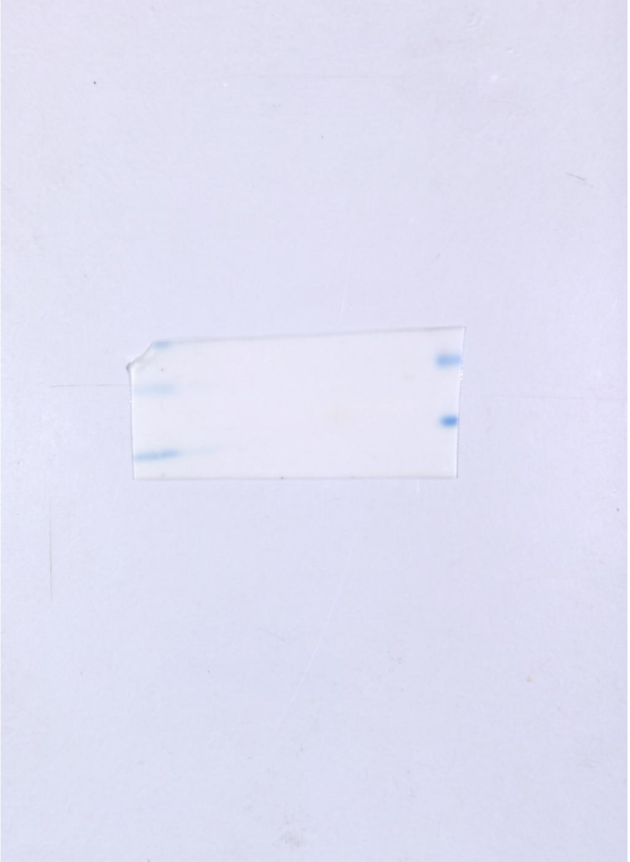

P21 M

Supplemental Material: The full length uncropped original western blots for Fig. 4C/DU 145

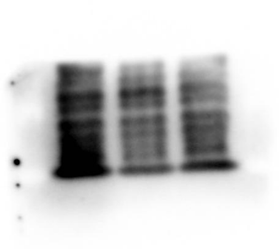

Survivin

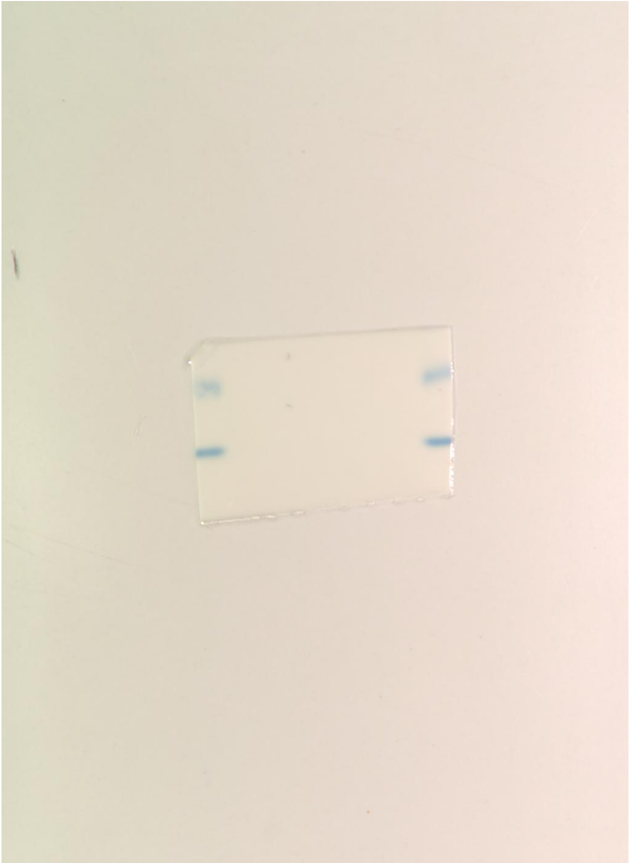

Survivin M

Supplemental Material: The full length uncropped original western blots for Fig. 4C/PC-3

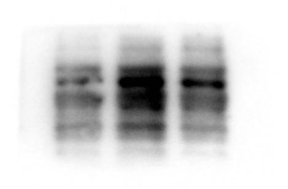

CD40L

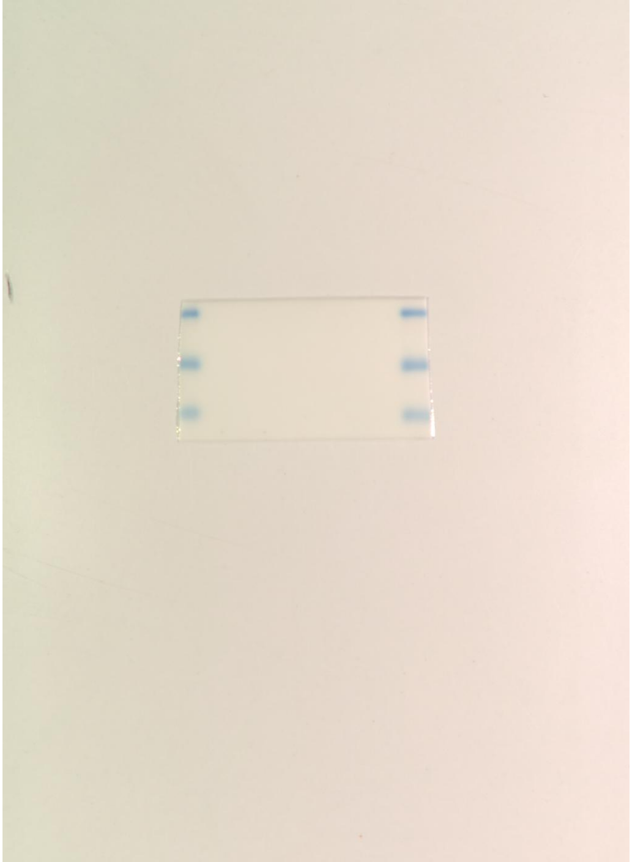

CD40L M

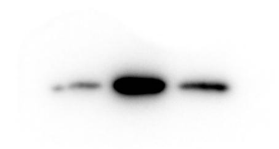

CYTC

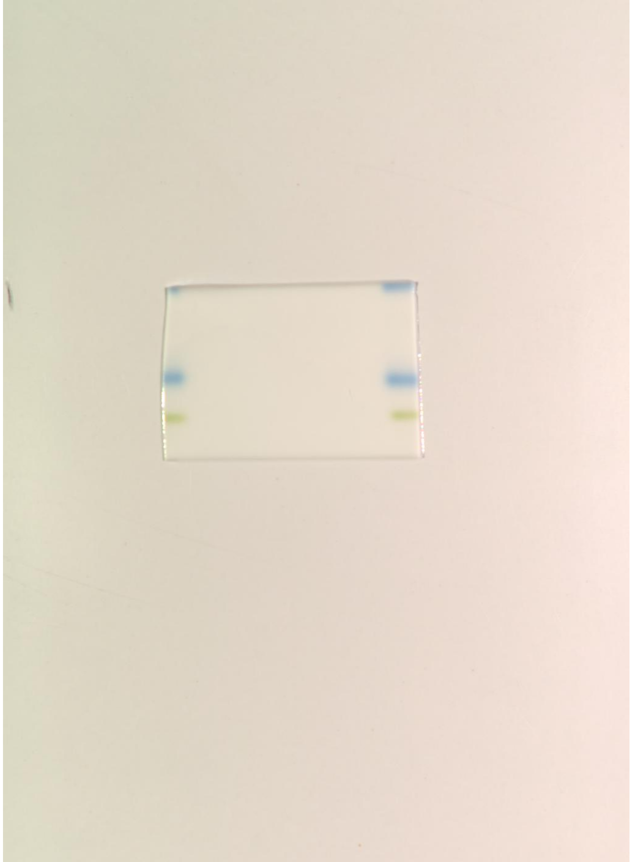

CYTC M

Supplemental Material: The full length uncropped original western blots for Fig. 4C/PC-3

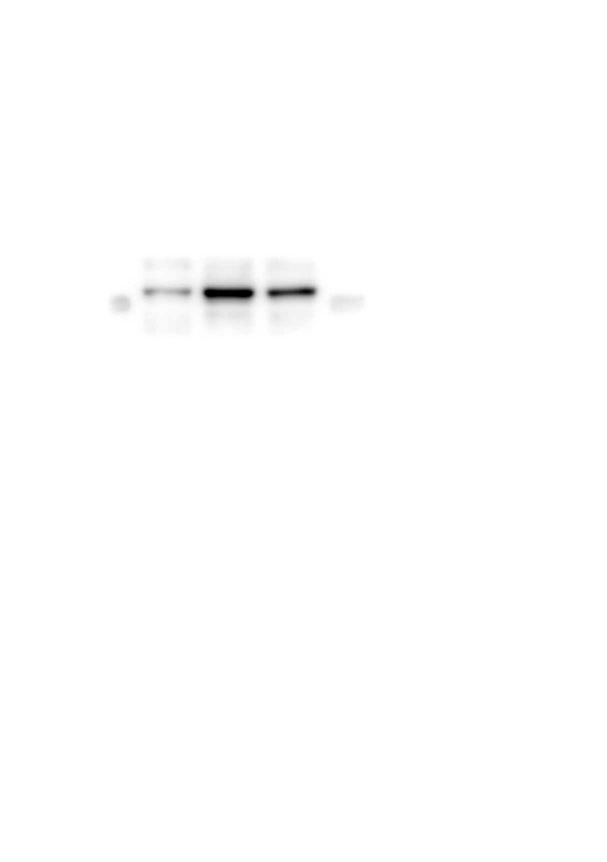

DR6

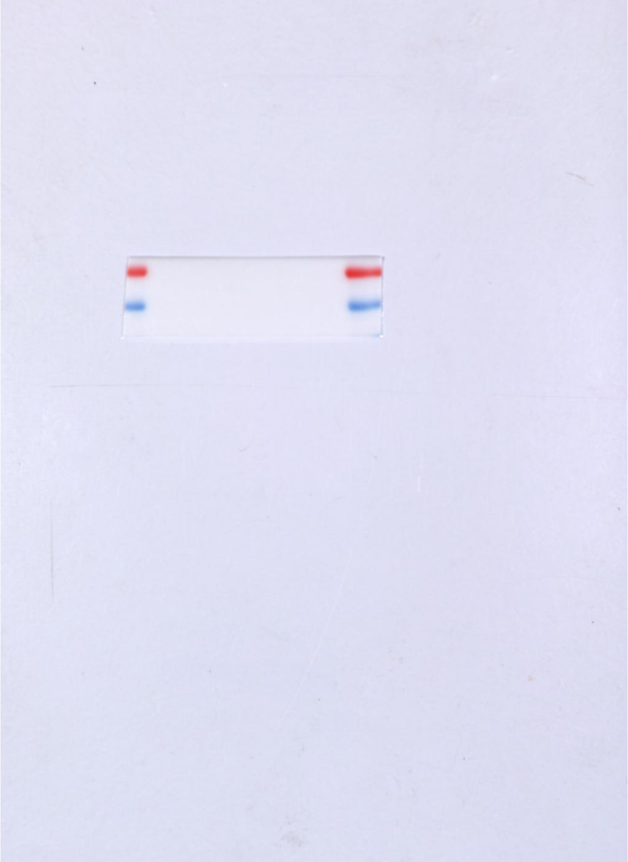

DR6 M

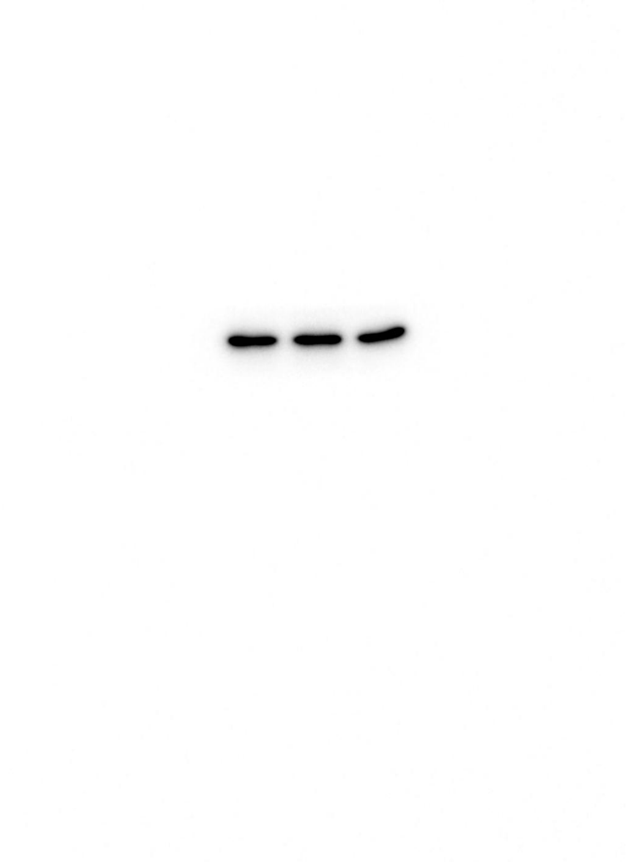

GAPDH

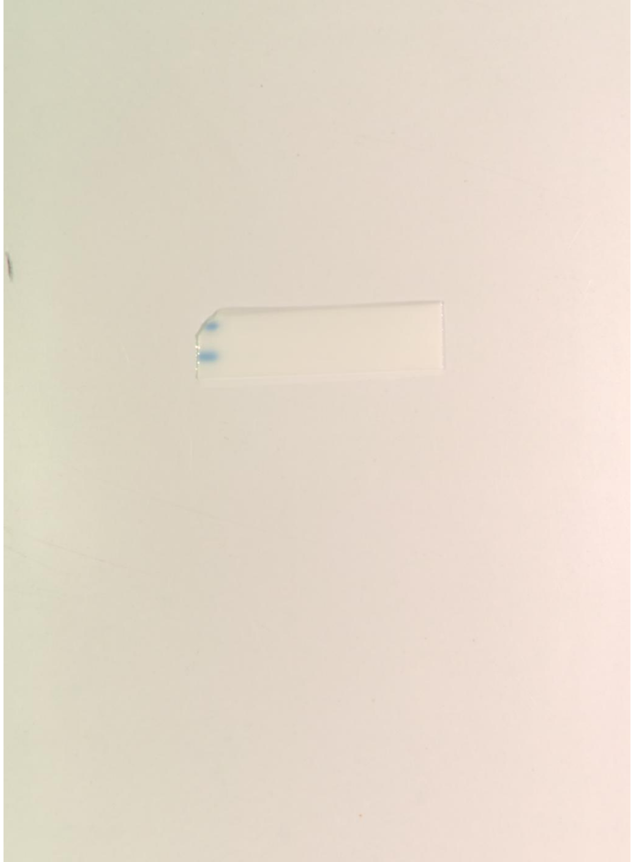

GAPDH M

Supplemental Material: The full length uncropped original western blots for Fig. 4C/PC-3

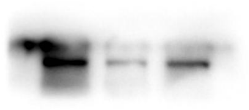

KIF15

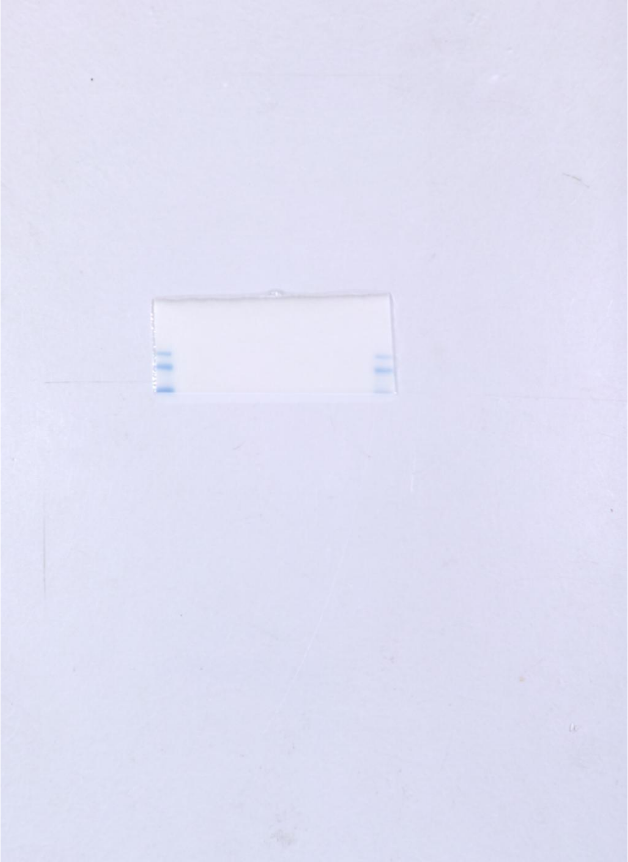

KIF15 M

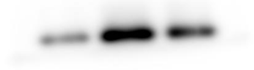

P21

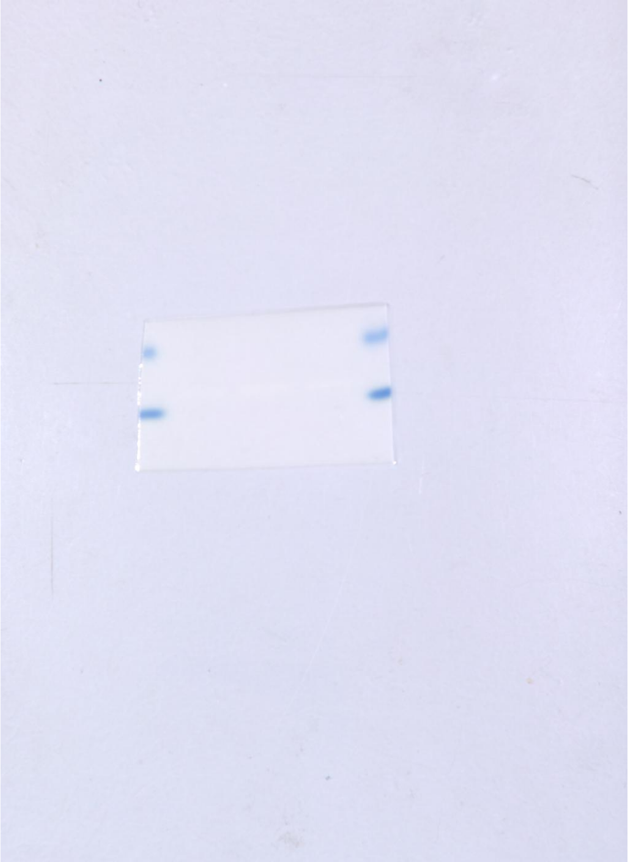

P21 M

Supplemental Material: The full length uncropped original western blots for Fig. 4C/PC-3

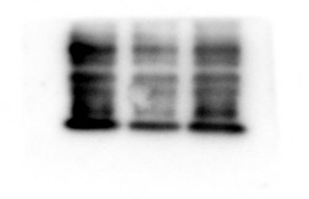

Survivin

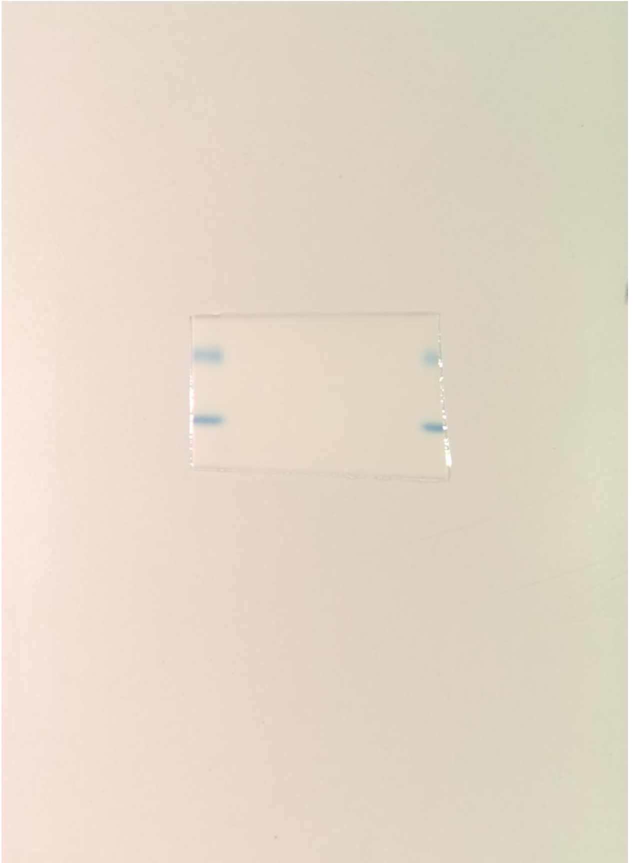

Survivin M

Supplemental Material: The full length uncropped original western blots for Fig. 5B

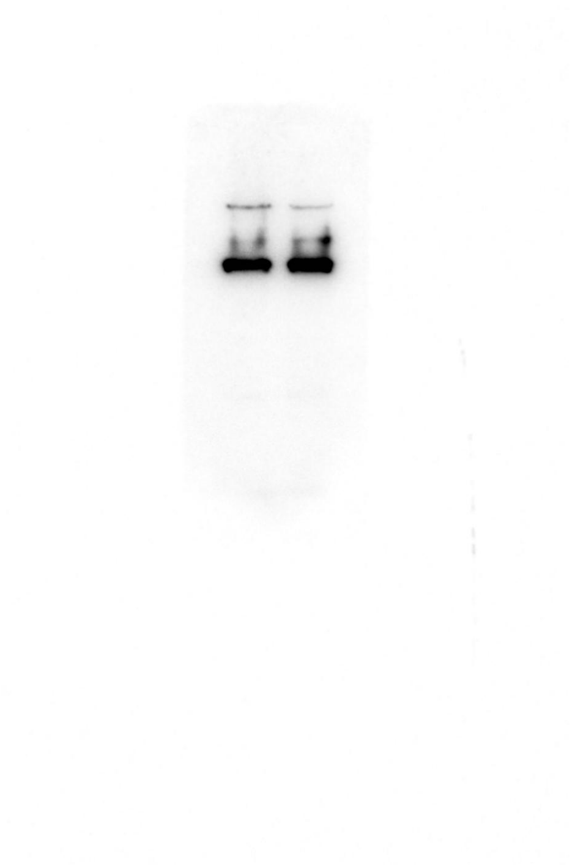

AKT

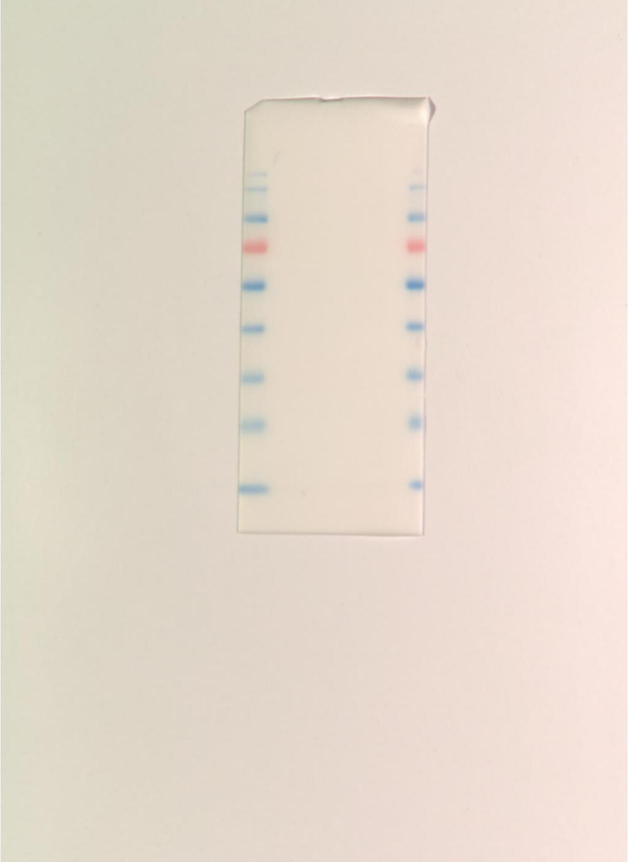

AKT M

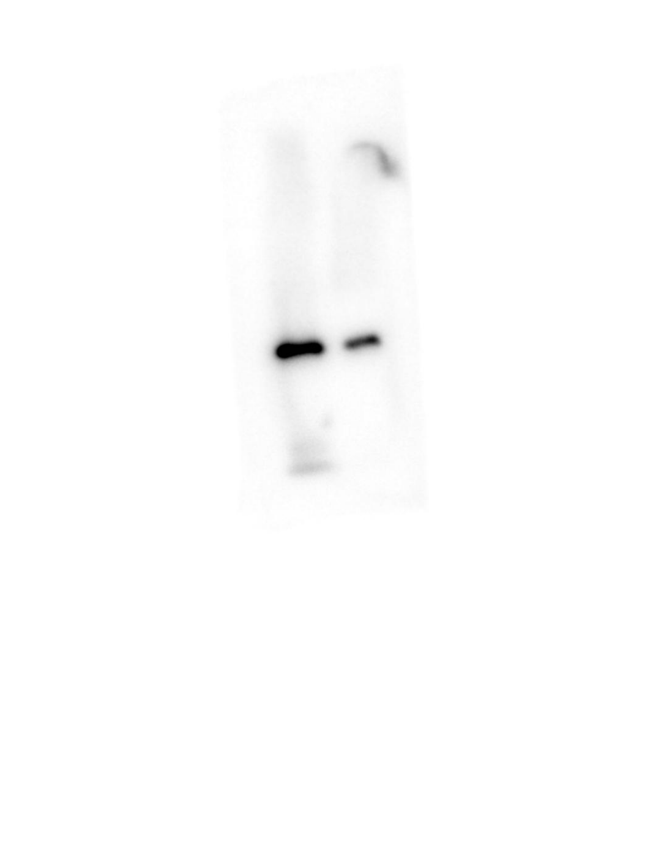

CCND1

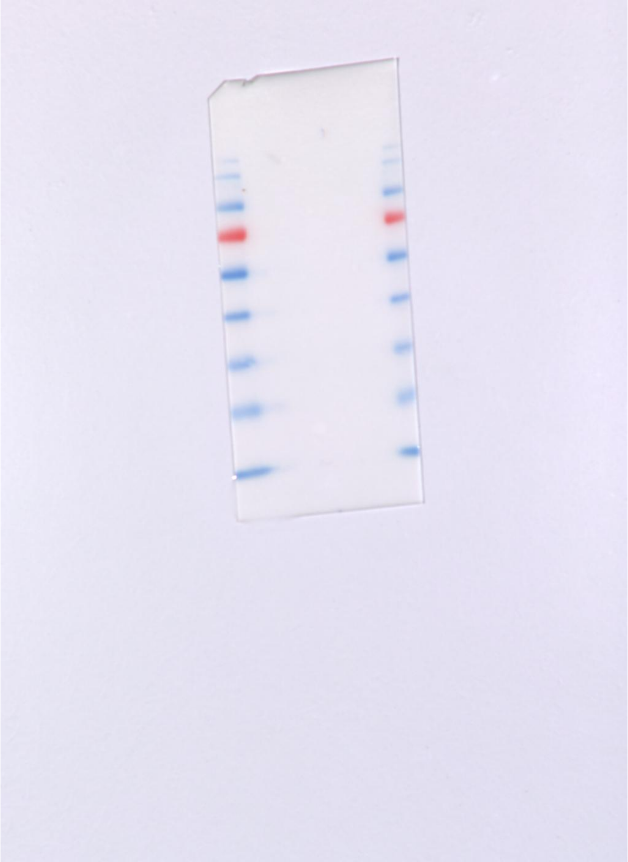

CCND1 M

Supplemental Material: The full length uncropped original western blots for Fig. 5B

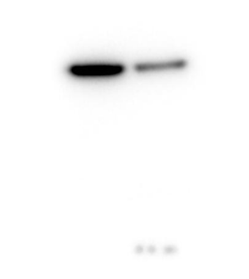

CDK6

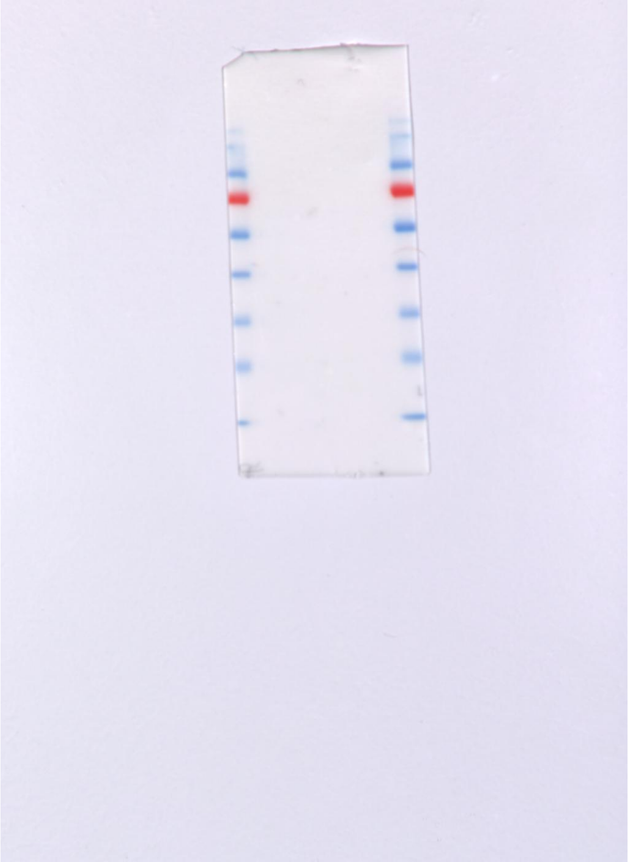

CDK6 M

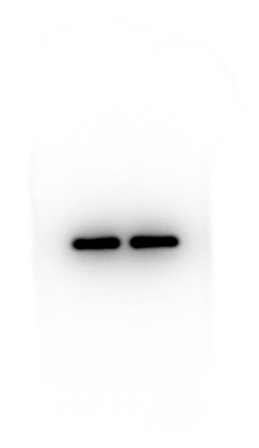

GAPDH

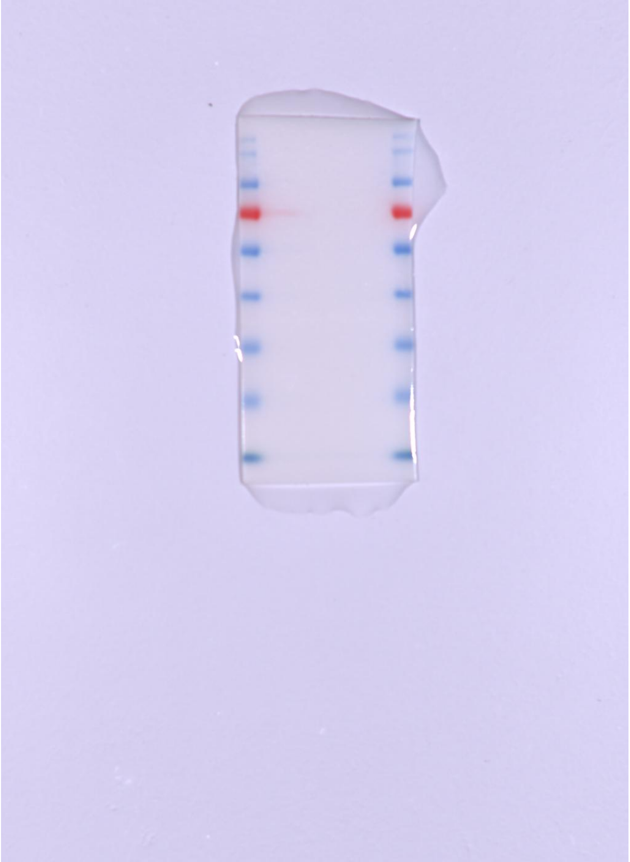

GAPDH M

Supplemental Material: The full length uncropped original western blots for Fig. 5B

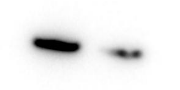

p-AKT

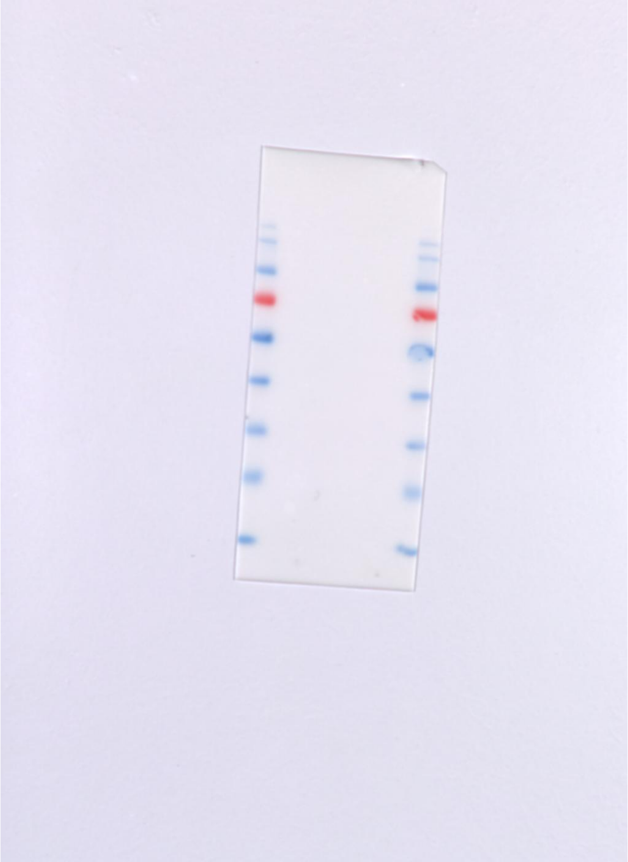

p-AKT M

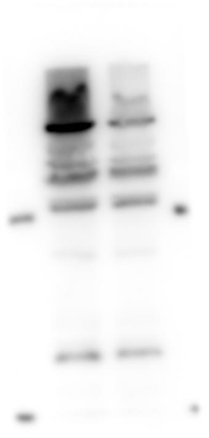

PIK3CA

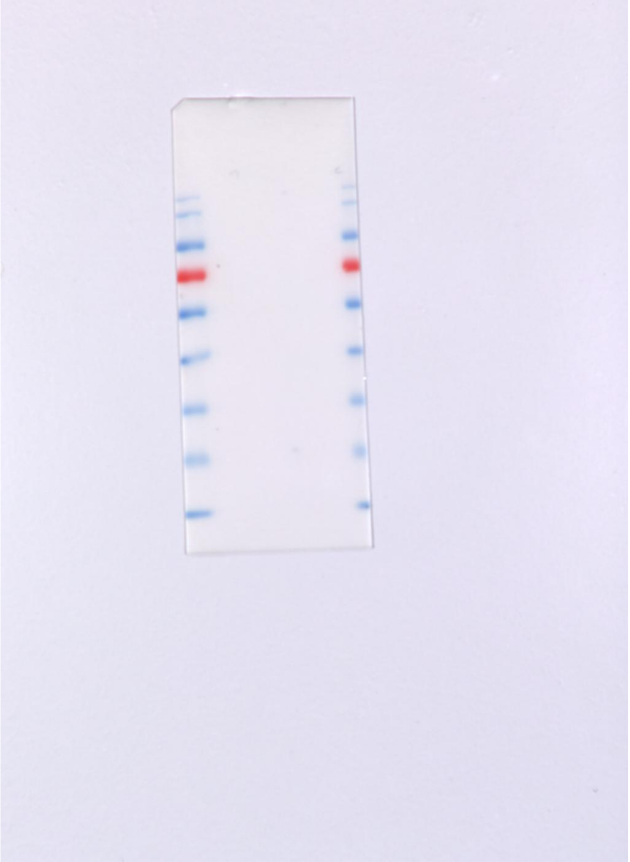

PIK3CA M

Supplemental Material: The full length uncropped original western blots for Fig. 5C

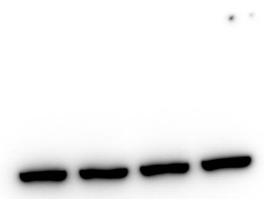

AKT

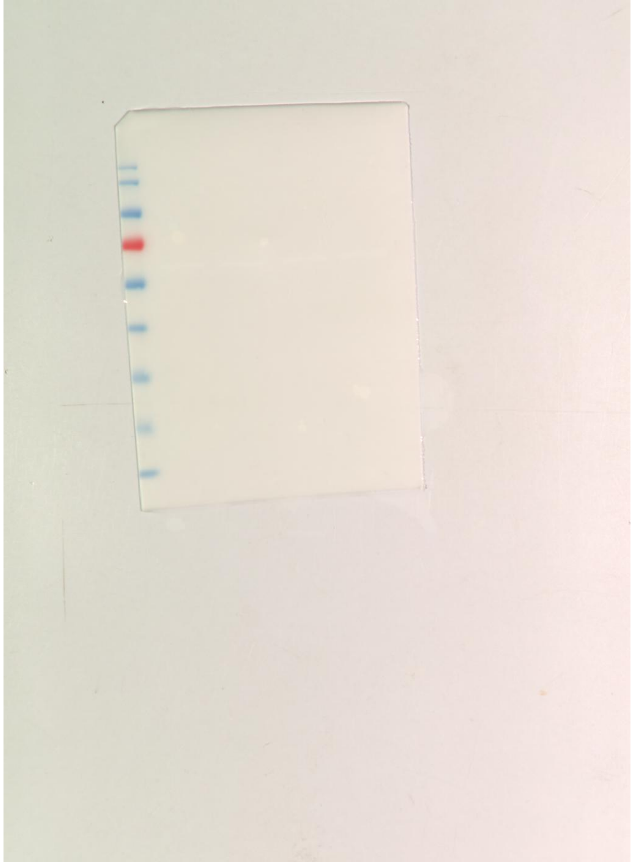

AKT M

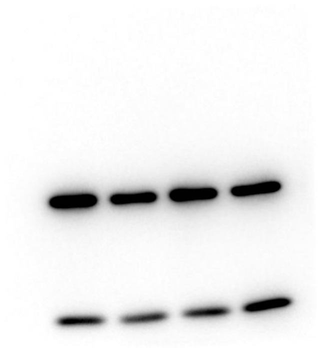

GAPDH

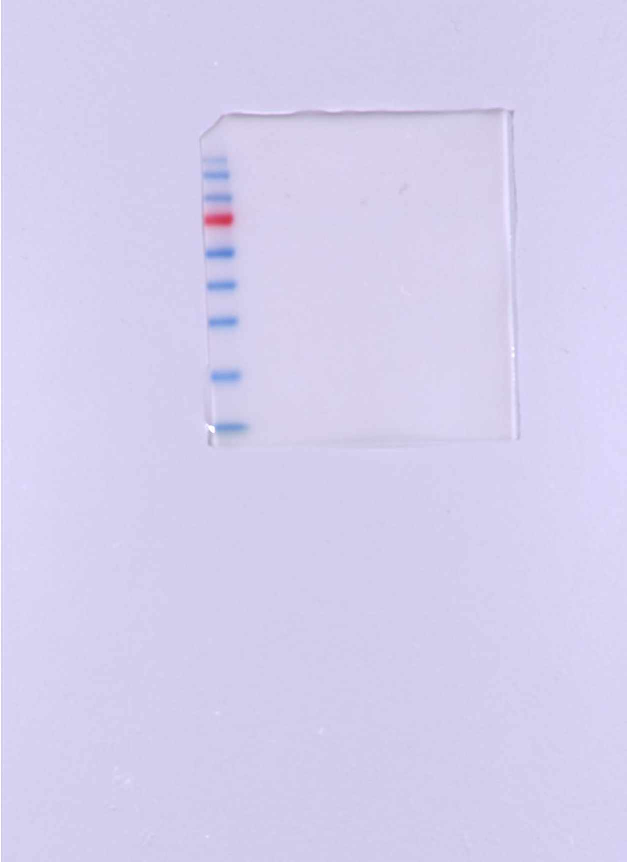

GAPDH M

Supplemental Material: The full length uncropped original western blots for Fig. 5C

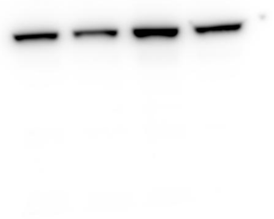

p-AKT

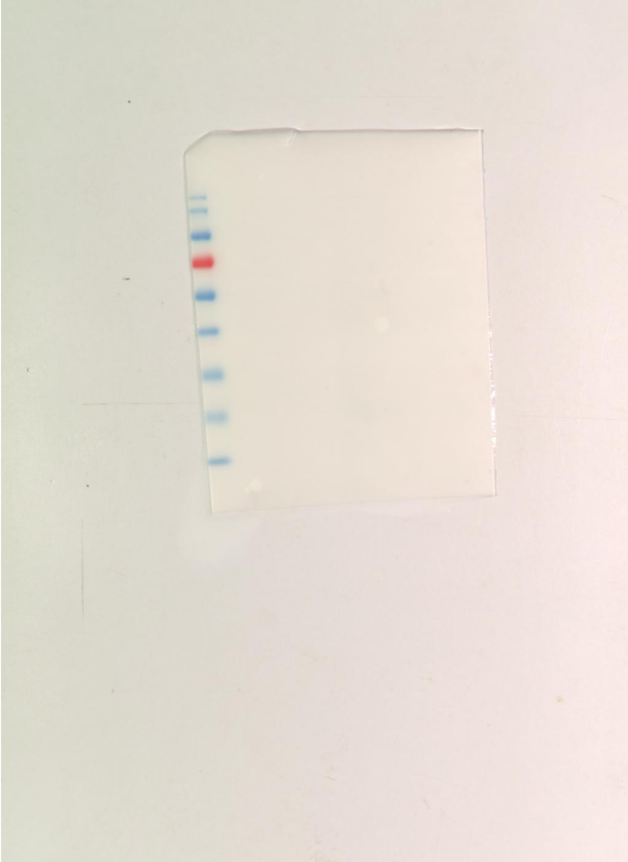

p-AKT M

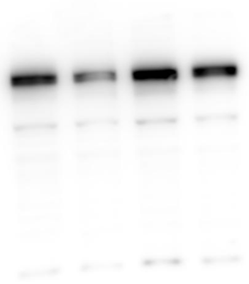

p-PI3K

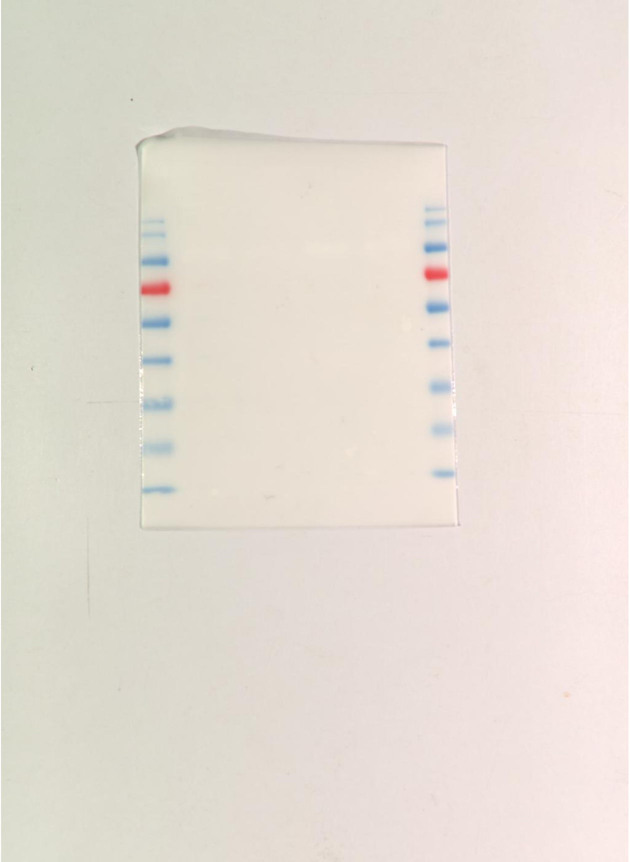

p-PI3K M

Supplemental Material: The full length uncropped original western blots for Fig. 5C

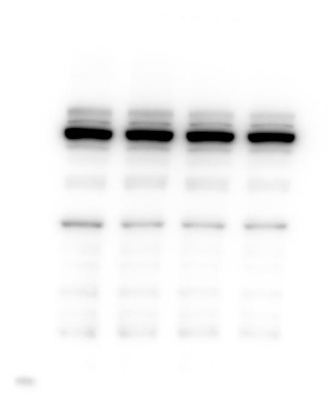

PI3K

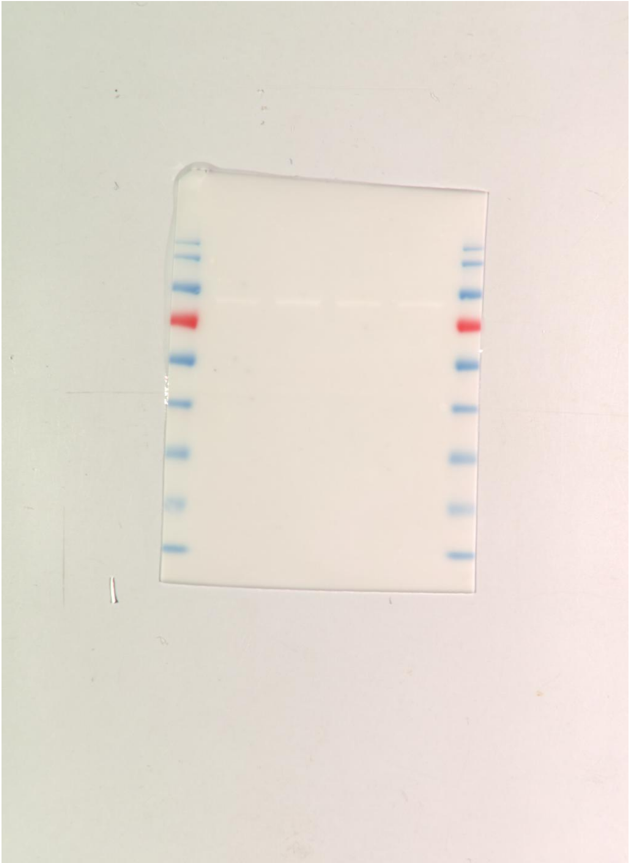

PI3K M

Supplemental Material: The full length uncropped original western blots for Fig. 6F

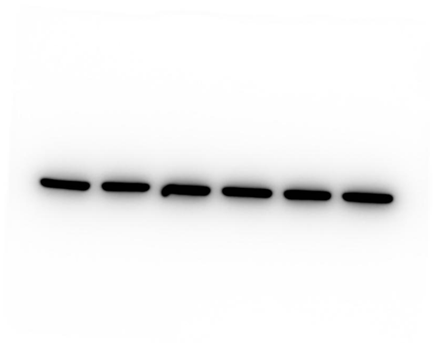

GAPDH

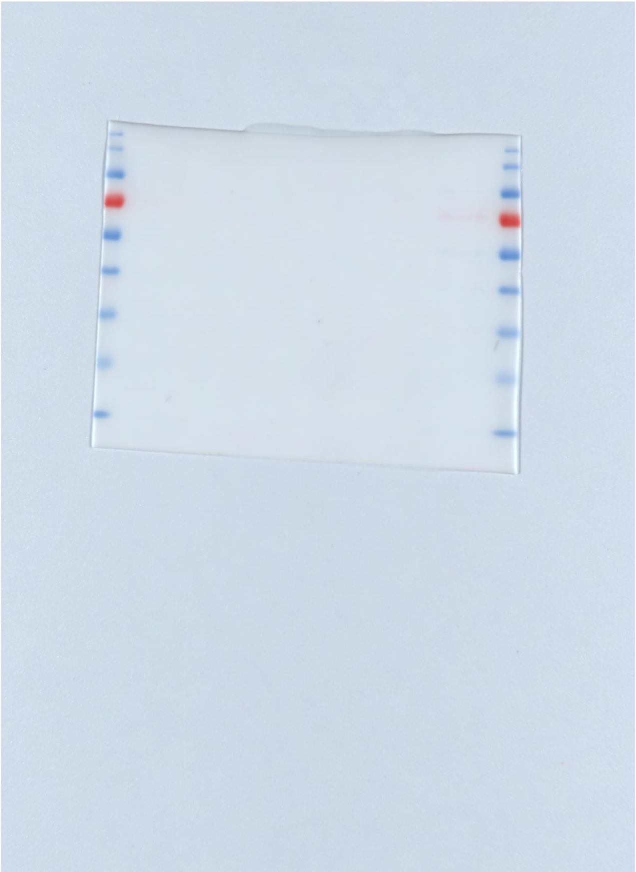

GAPDH M

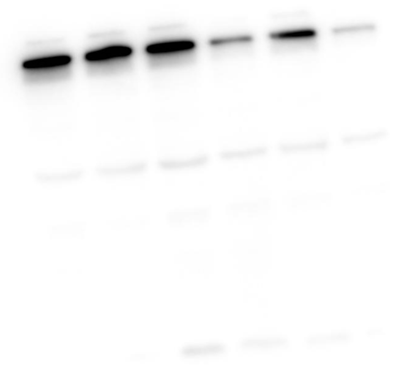

KIF15

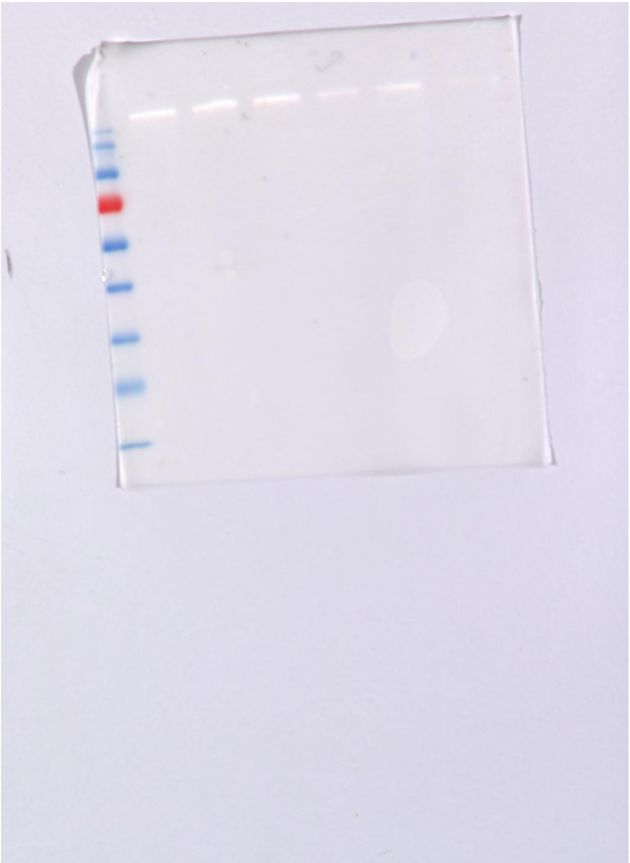

KIF15 M
